# HDAC6 controls innate immune and autophagy responses to TLR-mediated signalling by the intracellular bacteria *Listeria monocytogenes*

**DOI:** 10.1371/journal.ppat.1006799

**Published:** 2017-12-27

**Authors:** Olga Moreno-Gonzalo, Marta Ramírez-Huesca, Noelia Blas-Rus, Danay Cibrián, María Laura Saiz, Inmaculada Jorge, Emilio Camafeita, Jesús Vázquez, Francisco Sánchez-Madrid

**Affiliations:** 1 Cell-cell Communication Laboratory, Vascular Pathophysiology Area, Centro Nacional Investigaciones Cardiovasculares (CNIC), Madrid, Spain; 2 Servicio de Inmunología, Hospital Universitario de la Princesa, Instituto Investigación Sanitaria Princesa (IIS-IP)-Universidad Autónoma de Madrid (UAM), Madrid, Spain; 3 CIBER CARDIOVASCULAR, Madrid, Spain; 4 Proteomics Unit, Vascular Pathophysiology Area, Centro Nacional Investigaciones Cardiovasculares (CNIC), Madrid, Spain; University of Pennsylvania, UNITED STATES

## Abstract

Recent evidence on HDAC6 function underlines its role as a key protein in the innate immune response to viral infection. However, whether HDAC6 regulates innate immunity during bacterial infection remains unexplored. To assess the role of HDAC6 in the regulation of defence mechanisms against intracellular bacteria, we used the *Listeria monocytogenes* (*Lm*) infection model. Our data show that *Hdac6*^*-/-*^ bone marrow-derived dendritic cells (BMDCs) have a higher bacterial load than *Hdac6*^*+/+*^ cells, correlating with weaker induction of IFN-related genes, pro-inflammatory cytokines and nitrite production after bacterial infection. *Hdac6*^*-/-*^ BMDCs have a weakened phosphorylation of MAPK signalling in response to *Lm* infection, suggesting altered Toll-like receptor signalling (TLR). Compared with *Hdac6*^*+/+*^ counterparts, *Hdac6*^*-/-*^ GM-CSF-derived and FLT3L-derived dendritic cells show weaker pro-inflammatory cytokine secretion in response to various TLR agonists. Moreover, HDAC6 associates with the TLR-adaptor molecule Myeloid differentiation primary response gene 88 (*MyD88*), and the absence of HDAC6 seems to diminish the NF-κB induction after TLR stimuli. *Hdac6*^*-/-*^ mice display low serum levels of inflammatory cytokine IL-6 and correspondingly an increased survival to a systemic infection with *Lm*. The impaired bacterial clearance in the absence of HDAC6 appears to be caused by a defect in autophagy. Hence, *Hdac6*^*-/-*^ BMDCs accumulate higher levels of the autophagy marker p62 and show defective phagosome-lysosome fusion. These data underline the important function of HDAC6 in dendritic cells not only in bacterial autophagy, but also in the proper activation of TLR signalling. These results thus demonstrate an important regulatory role for HDAC6 in the innate immune response to intracellular bacterial infection.

## Author summary

*Listeria monocytogenes (Lm)* is a food-borne intracellular bacterium that causes listeriosis to 1.600 people each year, being responsible of approximately 260 deaths. This pathogen mostly affects immunocompromised individuals and pregnant women. It is particularly dangerous for the later due to its ability to pass across the placenta and the blood-brain barrier. *Lm* is extensively used as a *Gram* positive infection model in the laboratory to study innate and adaptive immune responses. HDAC6 is an important regulatory enzyme of the tubulin and actin cytoskeletons. Its inhibition or deficiency quells the immune response against different virus infections. Previous work has shown its involvement in the regulation of viral RNA-sensing activity and in interferon signalling. In this study, we report that HDAC6 is an essential component of the innate immune response to fight against intracellular bacterial infections. Genetic ablation of HDAC6 impairs activation of the pertinent Toll-like receptor pathway to induce the pro-inflammatory transcriptional program of the cell. Moreover, this enzyme controls cytoskeletal proteins that mediate the fusion of phagosome-contained bacteria with the lysosome during pathogen degradation.

## Introduction

Histone deacetylase 6 (HDAC6) is a cytoplasmic deacetylase involved in the regulation of several biological processes, including migration, transport, angiogenesis, and tumour progression [1–5]. This enzyme is able to deacetylate α-tubulin and cortactin, regulating not only the microtubule cytoskeleton, but also actin [6, 7]. Both cytoskeletal interactions underline a crucial role of HDAC6 in many cellular functions such as phagosome-lysosome fusion, cargo transport through microtubules, and cell motility [8–10]. The role of HDAC6 has also been described in two of the main cellular degradation mechanisms: autophagy, through interaction with the autophagy marker p62; and the proteasome, mediated by deacetylation of HSP90 and its intersection with the ubiquitin-proteasome system (UPS) [11–15]. In addition, HDAC6 is involved in the transport of damaged mitochondria (mitophagy) and misfolded proteins (aggrephagy) to lysosomes and the proteasome for degradation [16–18]. The absence of HDAC6 impairs the deacetylation of mitofusin 1, preventing the mitochondrial fusion induced by glucose deprivation and causing excessive ROS production that provokes oxidative damage [19].

HDAC6 regulates the replication of human immunodeficiency virus (HIV) by deacetylating Tat and thus inhibiting viral transactivation [20, 21]. HDAC6 also participates in Sendai virus infection through the deacetylation of β-catenin, which acts as a co-activator of IRF3-mediated transcription [22]. During infection with Influenza Virus A (IVA), HDAC6 appears to play a dual role. IVA capsids mimic misfolded-protein aggregates to take advantage of the host cell aggresome pathway, thereby achieving disassembly and successful viral uncoating [23]. On the other hand, HDAC6-mediated microtubule deacetylation impairs the IVA cycle, preventing trafficking of viral components to the viral assembly site in the host plasma membrane and the spread of infection to surrounding cells [24]. The role of HDAC6 in the adaptive CD4 + T-cell response has been studied in several autoimmune and inflammatory situations such as colitis and cardiac allograft rejection; however, little is known about its role in innate immunity and bacterial diseases [25, 26].

*Listeria monocytogenes* (*Lm*) is a *Gram*-positive bacteria that causes severe infection in immunocompromised individuals and is able to cross the blood-brain barrier and the placenta [27]. *Lm* is widely used as a model of innate and adaptive immune responses to intracellular bacterial infection [27–29]. From the first hours of infection, professional phagocytic cells trap bacteria in the blood and target organs, exerting a degree of control on bacterial growth [28]. After internalization by phagocytic cells, *Lm* is eliminated by fusion of the phagosome with lysosomes; however, some bacteria escape the phagosome into the cytoplasm through the action of listeriolysin O (LLO). In the cytoplasm, *Lm* replicates and is able to infect neighbouring cells [30–32]. Interestingly, phagosome-contained bacteria are also eliminated by the action of reactive oxygen species (ROS) and nitric oxide (NO), produced by NADPH oxidase 2 (NOX2) and inducible NO synthase (iNOS), respectively [33]. Moreover, *Lm* bacteria contain an ARP2/3-mimicking protein that enables their propulsion to neighbouring cells through the directional assembly of actin filaments (actin rockets) [34]. *Lm* can spread from cell to cell without exiting the intracellular compartment by a process called paracytophagy, which evades immune detection. However, the host cell is able to develop a specific CD8^+^T cell response to cytosolic *Lm*, which is crucial for the control of infection [35–38].

Early control of *Listeria* burden largely depends on the innate immune response occurring in the spleen, which relies on two main cell populations of dendritic cells (DCs). On one hand, a subset of monocyte-derived DCs namely TNF/iNOS–producing DCs (Tip-DCs) has the ability to produce TNFα and NO [39]. The other splenic DC subset is CD8α^+^ conventional DCs (cDCs), and it is responsible for the final resolution of infection against *Listeria* through the antigen presentation of bacterial-derived antigens to specific CD8+T cells to induce cytotoxicity [40, 41]. The response of dendritic cells (DC) to live *Lm* is mediated by toll-like receptors (TLRs), nucleotide-binding oligomerization domain (NODs)-like receptors (NLRs), and other cytosolic receptors and involves two signalling pathways: TLR-dependent and independent signalling. TLR-dependent signalling, triggered by sensing of cell-surface and endo-phagosomal bacteria, mediating the activation of a MyD88-dependent response; and the cytosolic pathway, triggered by bacterial DNA after the escape of *Lm* into the cytosol, is responsible for the activation of sensor stimulator of interferon (IFN) genes (STING). STING activation leads to IFN regulatory factor (IRF)3–dependent production of IFN-β and activation of downstream signals that control the transcription of IFN target genes essential for antiviral and antibacterial responses [42, 43].

To determine the role of HDAC6 in the innate response to bacterial infection, we explored the impact of HDAC6 deficiency on the response of myeloid cells to *Lm*. Our results reveal that *Hdac6*^*-/-*^ BMDCs are less efficient than *Hdac6*^*+/+*^ at clearing *Lm*. This is due to defective maturation of phagosome-contained bacteria. Moreover, *Hdac6*^*-/-*^ DCs display lower activation after *Lm* infection and TLR stimuli. These data support the view that HDAC6 positively regulates innate defence mechanisms against *Lm* and that its absence weakens the pro-inflammatory response.

## Results

### Deficient intracellular bacteria clearance in *Hdac6*^*-/-*^ BMDCs

**Fig 1:**
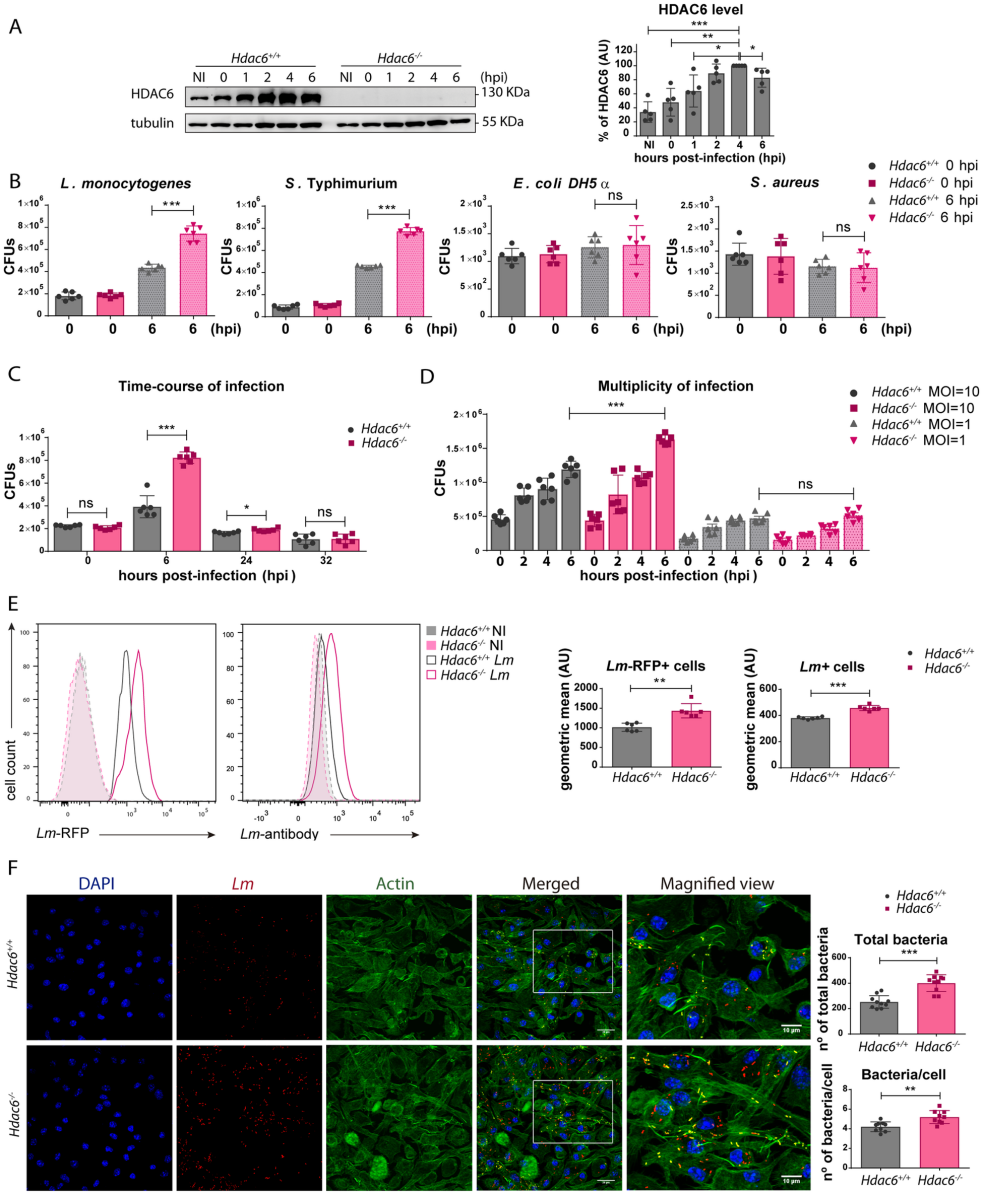
Deficient intracellular bacteria clearance in *Hdac6*^*-/-*^ BMDCs. A) Western blot analysis of HDAC6 in a time-course of infection of BMDCs with *Lm*. Tubulin was used as a loading control. HDAC6 levels were quantified in five independent experiments. ***p≤0.001, ** p≤0.01*p≤0.05. B) CFUs obtained at 0 and 6 hpi from BMDCs infected with *L*. *monocytogenes*, *S*. Typhimurium, *E*. *coli DHDα*, and *S*. *aureus* at a MOI of 10. Data from 0 hpi are shown as a bacteria entry control. ***p≤0.001, ns>0.05 non-significant; n = 6. C) CFUs of *Lm*-infected BMDCs obtained at 0, 6, 24 and 32 hpi with a MOI = 10. ***p≤0.001, *p≤0.05, ns>0.05 non-significant; n = 6. D) CFUs of *Lm*-infected BMDCs obtained at 0, 2, 4 and 6 hpi with a MOI = 10 and 1. ***p≤0.001, *p≤0.05, ns>0.05 non-significant; n = 6. E) BMDCs were infected with *Lm* or *Lm*-RFP for 6 h and the bacterial signal was determined by flow cytometry. The panel shows representative histograms and the geometric mean of the *Lm* signal. ***p≤0.001, ** p≤0.01; n = 6. F) Confocal microscopy determination of bacterial load at 6 hpi. *Left panel*: Maximum intensity z-projections of confocal microscopy images of *Lm*-infected *Hdac6*^*+/+*^ and *Hdac6*^*-/-*^ BMDCs at 6 hpi. The panel shows DAPI (blue), *Lm* (red), β-actin (green), merged views of three channels, and magnified views of the boxed areas from the merged view. Yellow indicates *Lm* and β-actin co-localization. Scale bars 20 μm (main panels) and 10 μm magnified views). *Right panel*: ImarisCell Module analysis of the number of cells and the number of bacteria per cell in all pictures (10 pictures per genotype). Statistical analysis of Imaris quantification of total bacteria and bacteria per cell in *Hdac6*^*+/+*^ and *Hdac6*^*-/-*^ BMDCs. ***p≤0.001, ** p≤0.01; n = 10.

To assess the possible role of HDAC6 in innate immune responses during bacterial pathogenesis, we performed a time-course infection with *Lm* in granulocyte and monocyte colony-stimulating factor (GM-CSF)-derived BMDCs from *Hdac6*^*+/+*^ and *Hdac6*^*-/-*^ mice. Increasing levels of HDAC6 expression were detected in the *Hdac6*^*+/+*^ DCs as the infection progressed (Fig 1A). However, BMDC differentiation was not noticeably affected in the absence of HDAC6 (S1 Fig part A). Next, *Hdac6*^*+/+*^ and *Hdac6*^*-/-*^ BMDCs were infected for different times with *Gram*-negative bacteria (*Salmonella* Typhimurium and *Escherichia coli DH5α*) and *Gram*-positive bacteria (*Listeria monocytogenes* and *Staphylococcus aureus*) at a multiplicity of infection (MOI) of 10, with colony-formed units (CFUs) corresponding to intracellular live bacteria. Bacterial entry was similar in *Hdac6*^*+/+*^ and *Hdac6*^*-/-*^ DCs at 0 h post-infection (hpi), while bacterial proliferation, measured at 6 hpi, was significantly higher in *Hdac6*^*-/-*^ BMDCs for both types of intracellular pathogens, *Lm* and *S*. Typhimurium (Fig 1B). This was not due to differences in cell viability at 6 hpi (S1 Fig part B). In contrast, no significant difference was observed in the proliferation of the non-intracellular pathogens *S*. *aureus* and *E*. *coli*, indicating that HDAC6 is an important component of cellular mechanisms for the clearance of intracellular pathogens (Fig 1B).

Time-course analysis showed that differences between *Lm* infection in *Hdac6*^*+/+*^ and *Hdac6*^*-/-*^ BMDCs CFUs peaked at 6 hpi and were sustained until 24 hpi (Fig 1C). This effect was clearly observed at a MOI of 10, which did not affect cell viability (Fig 1D and S1 Fig part B). A similar pattern was observed with macrophage colony-stimulating factor (M-CSF)-derived macrophages, demonstrating the lineage independence of the role of HDAC6 in bacterial clearance (S1 Fig part C). Although the difference between *Hdac6*^*+/+*^ and *Hdac6*^*-/-*^ cells was observed in both macrophages and DCs, the clearance capacity of macrophages was ten-fold higher than that of DCs at 6 hpi (S1 Fig part C).

Bacterial load was also determined by flow cytometry using two strategies: a specific antibody against *Lm*, and RFP-expressing bacteria. Both approaches showed that *Hdac6*-deficient DCs contained more bacteria at 6 hpi (Fig 1E). Higher numbers of bacteria in *Hdac6*^*-/-*^ BMDCs were also detected by confocal fluorescence microscopy at 6 hpi (Fig 1F). Some bacteria co-localized with filamentous actin, showing clear actin rockets (Fig 1F). Image quantification confirmed that *Hdac6*^*-/-*^ BMDCs contained more bacteria per cell and more total bacteria, remarking a higher percentage of cells hosting a large number of bacteria in *Hdac6*^*-/-*^ cells (see distribution of bacteria per cell, 6–7) (S1 Fig part D). ImarisCell Module view of Fig 1F images showed the number of bacteria per cell using actin transparency to easily visualize individual bacteria (S1 Fig part E).

**Fig 2:**
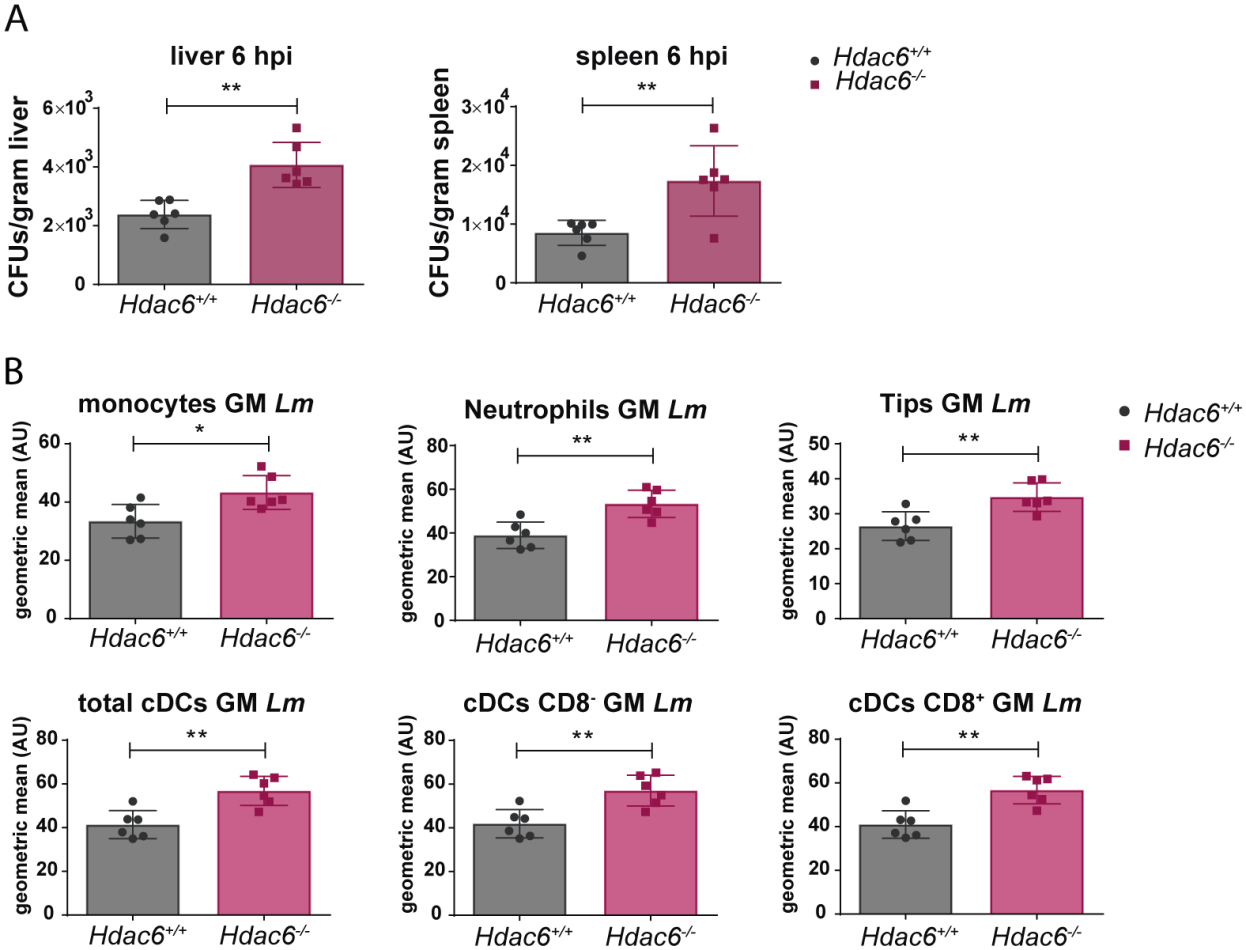
Deficient intracellular bacteria clearance in *Hdac6*^*-/-*^ splenic myeloid populations. A) Quantification of bacterial load in target organs (spleen and liver) at 6 hpi in *Hdac6*^*+/+*^ and *Hdac6*^*-/-*^ mice injected with a lethal dose of *Lm*. Bacterial load is expressed by CFUs per gram of liver (left graph) and per gram of spleen (right graph). **p≤0.01, n = 6. B) The charts show geometric means of *Lm* of different splenic populations (monocytes, neutrophils, Tips DCs, total cDCs, cDCs CD8^-^ and cDCs CD8^+^) gated in the live CD3^-^CD19^-^DX5^-^ population of *Hdac6*^*+/+*^ and *Hdac6*^*-/-*^ mice injected with a lethal dose of *Lm* at 6 hpi. **p≤0.01; n = 6.

To ascertain whether *Hdac6*^*-/-*^ cells display higher bacterial burden than *Hdac6*^*+/+*^ cells *in vivo*, *Hdac6*^*+/+*^ and *Hdac6*^*-/-*^ mice were intravenously injected with *Lm* and total CFUs per gram of liver and spleen were determined at 6 hpi. In agreement with the higher numbers of *Lm* observed in GM-CSF-DCs and M-CSF-Macrophages, we observed increased bacterial titres in spleen and liver cell suspensions (Fig 2A). Next, to determine the specific cell populations underlying this phenotype, a multicolour gating strategy was used to identify the myeloid cell compartment, including monocytes, neutrophils, Tips DCs, total cDCs, cCDs CD8^-^ and cDCs CD8^+^ (S2 Fig part A). Higher numbers of *Lm* were observed in different myeloid cells at 6 hpi (Fig 2B and S2 Fig part B). These data highlight the impairment of *Hdac6*^*-/-*^ myeloid cells to clear intracellular *Lm*.

### Impaired bacterial clearance in *Hdac6*^*-/-*^ BMDCs is caused by a defect in autophagy

**Fig 3:**
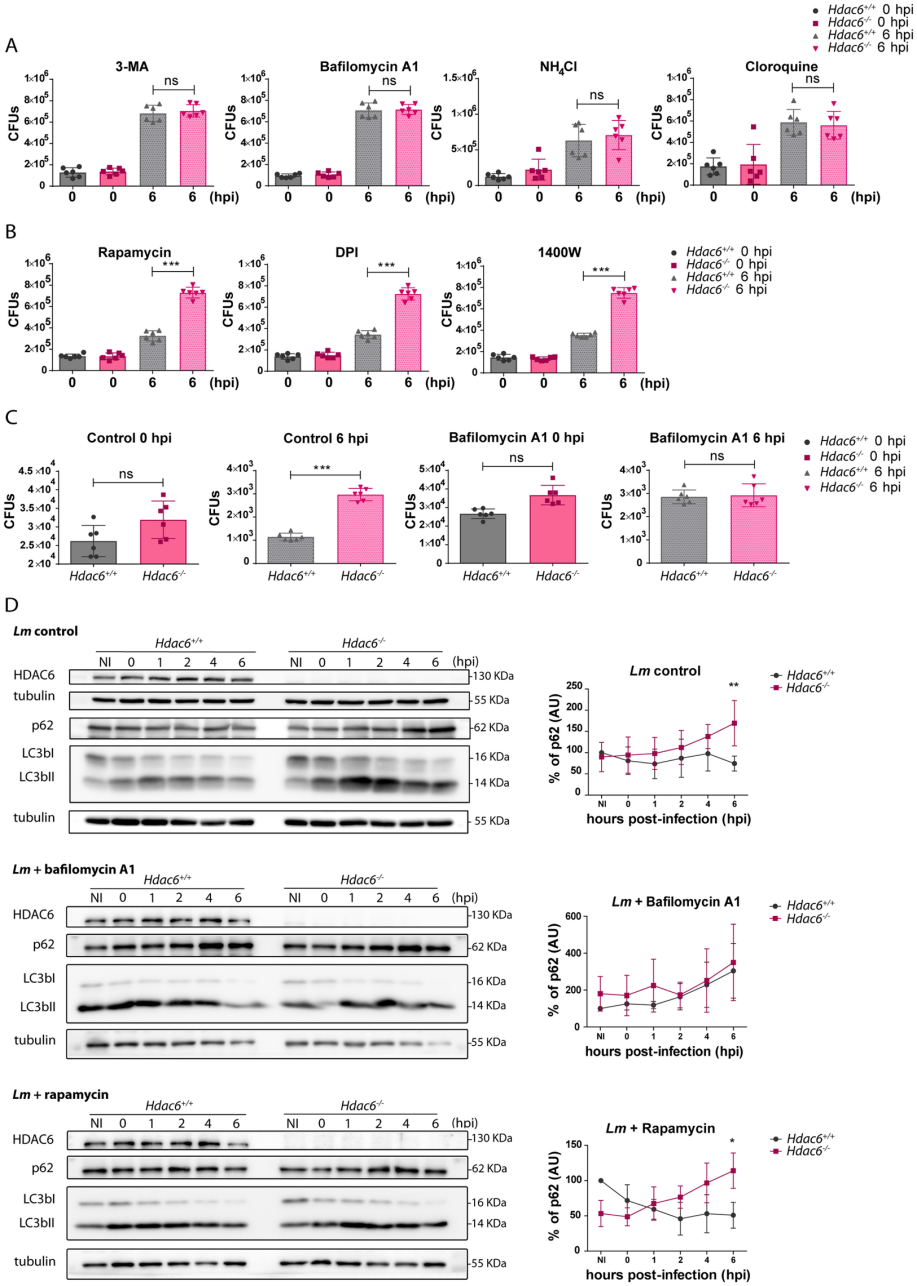
Impaired bacterial clearance in *Hdac6*^*-/-*^ BMDCs is caused by a defect in autophagy. A) Total CFUs in *Lm*-infected BMDCs treated with inhibitors. CFUs were detected at entry (0 hpi) and 6 hpi (bacterial proliferation) using the autophagy inhibitors (3-MA and bafilomycin A1 and the lysosome acidification inhibitors (NH4Cl and cloroquine), ns>0.05 non-significant; n = 6. B) Total CFUs at 0 and 6 hpi in *Lm*-infected BMDCs treated with the autophagy activator (rapamycin), the NADPH oxidase inhibitor (DPI) and the iNOS inhibitor (1400W). ***p≤0.001; n = 6. C) Total CFUs at 0 and 6 hpi in *Lm*-infected thioglycollate-elicited macrophages treated with or without bafilomycin A1. ***p≤0.001, ns>0.05 non-significant; n = 6. D) Western-blot analysis of autophagy markers over the time-course of *Lm* infection in *Hdac6*^*+/+*^ and *Hdac6*^*-/-*^ BMDCs. *Left panels*: Levels were detected of p62, LC3bI and II and HDAC6 in control cells and cells treated with bafilomycin A1 and rapamycin. Tubulin was used as a loading control. HDAC6 was as a genotype check of *Hdac6*^*+/+*^ and *Hdac6*^*-/-*^ BMDCs and to monitor HDAC6 induction during infection. *Right panels*: Accompanying charts show quantification of the p62 percentage of control, bafilomycin A1 and rapamycin western blots. ** p≤0.01, * p≤0.05, ns>0.05 non-significant; n = 5.

To test the involvement of autophagy in the mechanism by which HDAC6 regulates *Lm* infection, we treated DCs with 3-methyladenine (3-MA), an inhibitor of autophagosome formation. Treatment with 3-MA increased bacterial load in *Hdac6*^*+/+*^ BMDCs at 6 hpi, while having no effect on *Hdac6*^*-/-*^ BMDCs (Fig 3A), suggesting autophagy as the bacterial clearance mechanism impaired in *Hdac6*-deficient DCs. A similar result was observed upon treatment of BMDCs with bafilomycin A1, an inhibitor of vascular proton pump that indirectly inhibits phagosome-lysosome fusion, and with the lysosome acidification inhibitors chloroquine and NH_4_Cl (Fig 3A). In contrast, increasing autophagy flux with rapamycin did not restore the impaired autophagy in *Hdac6*^*-/-*^ BMDCs (Fig 3B). No significant effects were observed with control vehicles (S3 Fig part A). To explore other possible mechanisms, we treated BMDCs with inhibitors of NADPH oxidase (DPI) and iNOS (1400W). These treatments did not alter the difference in CFU number at 6 hpi between treated and non-treated *Hdac6*^*+/+*^ and *Hdac6*^*-/-*^ BMDCs, indicating that the activity of either enzyme is not accounting for the existing phenotype (Fig 3B).

The defective autophagy phenotype of *Hdac6*^*-/-*^ BMDCs was not due to transcriptional alterations to autophagy or lysosome components, since *Lm*-infected *Hdac6*^*+/+*^ and *Hdac6*^*-/-*^ BMDCs showed no mRNA expression differences at 6 hpi in the autophagy components LC3A and B, p62, ATG2, 5, 7 and 12, and Beclin-1 or in the lysosome components LAMP-1 and 2 (S3 Fig part B).

To determine whether these findings can be extended to other phagocytic cells, we carried out CFU assays with macrophages obtained from *Hdac6*^*+/+*^ and *Hdac6*^*-/-*^ mice four days after intraperitoneal thioglycollate injection. Higher bacterial load was observed only in *Hdac6*^*-/-*^ macrophages at 6 hpi, and this difference was abrogated by treatment with bafilomycin A1 (Fig 3C). These data indicate that the phenotype observed in BMDCs is also extendable in other *Hdac6*-deficient phagocytic cells such as peritoneal macrophages, indicating a widespread defect in intracellular killing ability due to lack of HDAC6. Moreover, the killing ability shown by peritoneal macrophages is similar to that of M-CSF-derived macrophages and higher than GM-CSF-derived DCs (Fig 3C compared with S1 Fig part C).

To gain further insight into the autophagy mechanism affected by HDAC6, we monitored the autophagosome markers p62 and LC3bI and II in *Lm*-infected BMDCs. *Hdac6*^*-/-*^ BMDCs showed a 2-fold higher accumulation of p62 than *Hdac6*^*+/+*^ cells at 6 hpi and increased LC3bII level in *Hdac6*^*+/+*^ cells from 1 to 6 hpi (Fig 3D). However, differences in p62 and LC3b levels were not noticed at early times of *Lm* infection of *Hdac6*^*+/+*^ and *Hdac6*^*-/-*^ BMDCs, indicating that the induction of autophagy is not affected in the absence of HDAC6 (Fig 3D). The treatment with bafilomycin A1 enhances the accumulation of p62 during the infection at the same level in both genotypes, abrogating the deficiency in autophagy observed in *Hdac6*^*+/+*^ BMDCs (Fig 3D). Although rapamycin also increased p62 accumulation at early times in *Hdac6*^*+/+*^ and *Hdac6*^*-/-*^ BMDCs, only *Hdac6*^*+/+*^ cells are able to diminished p62 at 6 hpi (Fig 3D). This treatment confirmed the results obtained in the CFUs functional assays with this inhibitor (Fig 3D compared with Fig 3B). The similarity of the autophagy defect detected in *Hdac6*^*-/-*^ BMDCs in control condition to that in rapamycin-treated *Hdac6*^*-/-*^ cells, suggests an impairment in phagocytic vesicle fusion with the lysosome.

### *Hdac6*^*-/-*^ BMDCs accumulate p62

**Fig 4:**
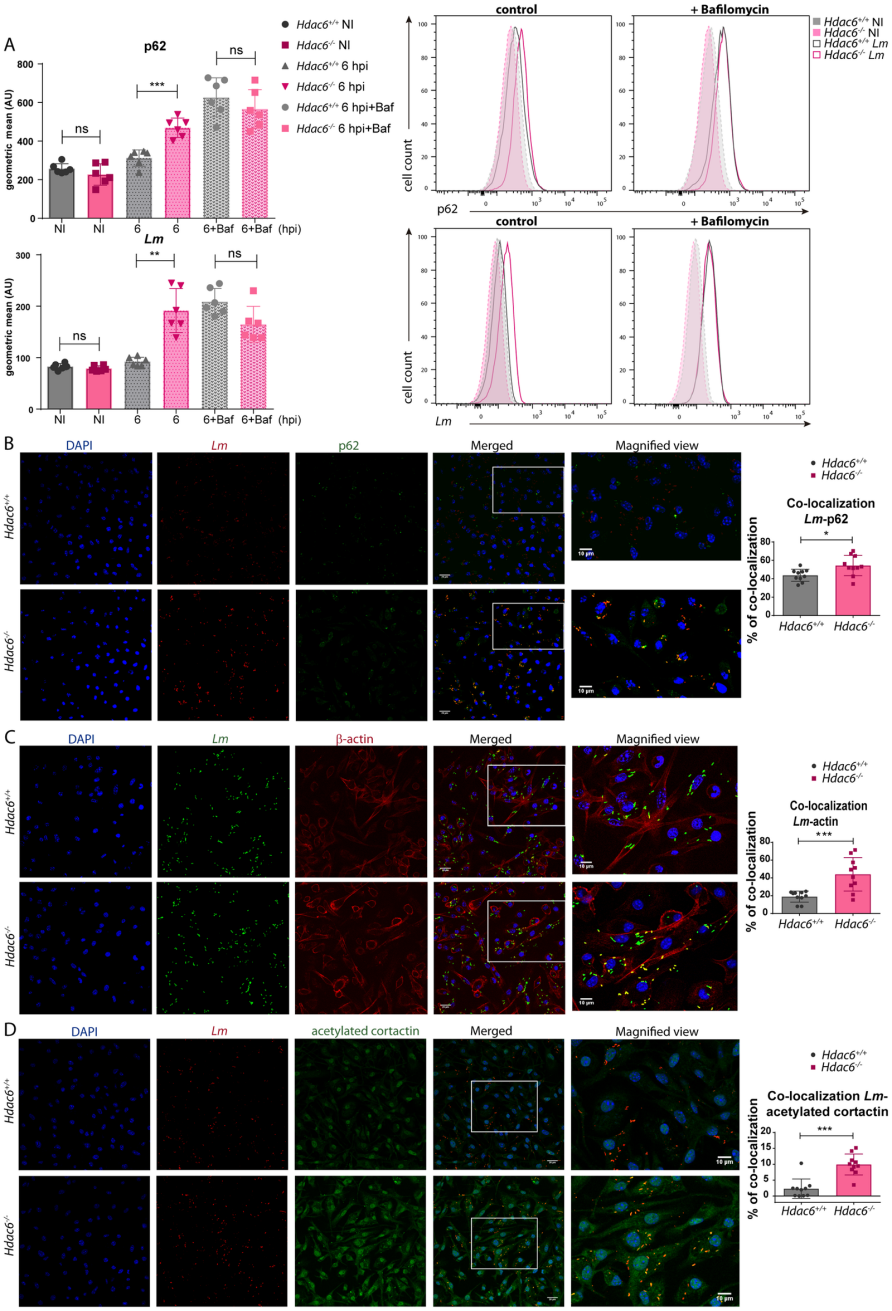
*Hdac6*^*-/-*^ BMDCs accumulate higher levels of p62. A) *Left panels*: The charts show geometric means of p62 *and Lm* gated in the MHCII^+^CD11c^+^ population of *Hdac6*^*+/+*^ and *Hdac6*^*-/-*^ BMDCs without infection (NI) and at 6 hpi, with and without bafilomycin A1 treatment. The representative histograms on the right show p62 and *Lm* with and without bafilomycin A1. ***p≤0.001, ** p≤0.01, ns>0.05 non-significant; n = 6. B) Confocal microscopy analysis of p62-*Lm* co-localization in *Lm*-infected *Hdac6*^*+/+*^ and *Hdac6*^*-/-*^ BMDCs at 6 hpi. Panels show DAPI (blue), *Lm* (red), p62 (green), and merged views of the three channels, with magnified views of the boxed areas. Yellow indicates p62-*Lm* co-localization. Scale bars 20 μm (main panels) and 10 μm (magnified views). *Right panel*: The chart shows ImarisCell Module analysis of the number of cells and the number of bacteria per cell in all pictures (10 pictures per genotype). Co-localization percentages were obtained by measuring the p62 channel on the bacterial surface using a threshold of 100. The statistical analysis of Imaris quantifications corresponds to the percentage of p62-*Lm* co-localization at 6 hpi. *p≤0.05; n = 10. C) Confocal microscopy analysis of actin-*Lm* co-localization in *Lm*-infected *Hdac6*^*+/+*^ and *Hdac6*^*-/-*^ BMDCs at 6 hpi. Panels show DAPI (blue), *Lm* (green), β-actin (red), and merged views of the three channels, with magnified views of the boxed areas. Yellow indicates β-actin-*Lm* co-localization. Scale bars 20 μm (main panels) and 10 μm (magnified views). *Right panel*: The chart shows ImarisCell Module analysis of the number of cells and the number of bacteria per cell in all pictures (10 pictures per genotype). Co-localization percentages were obtained by measuring the actin channel on the bacterial surface using a threshold of 40.6. The statistical analysis of Imaris quantifications corresponds to the percentage of actin-*Lm* co-localization at 6 hpi. *** p≤0.001; n = 10. D) Confocal microscopy analysis of acetylated cortactin-*Lm* co-localization in *Lm*-infected *Hdac6*^*+/+*^ and *Hdac6*^*-/-*^ BMDCs at 6 hpi. Panels show DAPI (blue), *Lm* (red), acetylated cortactin (green), and merged views of the three channels, with magnified views of the boxed areas. Yellow indicates acetylated cortactin-*Lm* co-localization. Scale bars 20 μm (main panels) and 10 μm (magnified views). *Right panel*: The chart shows ImarisCell Module analysis of the number of cells and the number of bacteria per cell in all pictures (10 pictures per genotype). Co-localization percentages were obtained by measuring the acetylated cortactin channel on the bacterial surface using a threshold of 184. The statistical analysis of Imaris quantifications corresponds to the percentage of acetylated cortactin-*Lm* co-localization at 6 hpi. *** p≤0.001; n = 10.

In order to further understand the defective autophagy of *Hdac6*^*-/-*^ BMDCs, the accumulation of p62 was studied in more detail. Flow cytometry at 6 hpi revealed significantly higher p62 content in *Hdac6*^*-/-*^ BMDCs, indicating accumulation of this phagosome marker due to defective fusion of this organelle with the lysosome (Fig 4A). Bafilomycin A1 treatment completely abrogated this difference, suggesting that *Hdac6*^*-/-*^ BMDCs displayed an impairment in the final step of autophagy (Fig 4A). More signal of *Lm* is displayed in *Hdac6*^*-/-*^ DCs (Fig 4A). In this regard, bafilomycin A1 treatment increased the low *Lm* signal in *Hdac6*^*+/+*^ DCs to the level observed in *Hdac6*^*-/-*^ cells (Fig 4A).

Confocal fluorescent analysis of *Lm*-infected DCs revealed increased levels of p62 in *Hdac6*^*-/-*^ BMDCs (Fig 4B). *Hdac6*^*-/-*^ BMDCs also showed a higher percentage of p62-*Lm* co-localization than *Hdac6*^*+/+*^ cells, indicating that *Hdac6*^*-/-*^ cells have more number of phagosome-contained bacteria (Fig 4B in accordance with p62 accumulation observed in Figs 3D and 4A). Confocal fluorescent microscopy study of actin and *Lm* revealed more frequent co-localization in *Hdac6*^*-/-*^ than in *Hdac6*^*+/+*^ BMDCs, indicating that more bacteria are at the cytoplasm to form actin rockets in *Hdac6*-deficient cells (Fig 4C). Moreover, more signal of acetylated-cortactin is detected in *Hdac6*^*-/-*^ BMDCs and also higher percentage of acetylated-cortactin-*Lm* co-localization (Fig 4D). These data could explain the accumulation of p62 and the delay in phagocytic vesicle fusion observed in *Hdac6*^*-/-*^ BMDCs, necessary to degrade phagocytosed *Lm*.

### Defective pro-inflammatory cytokine response to *Lm* in *Hdac6*^*-/-*^ BMDCs

**Fig 5:**
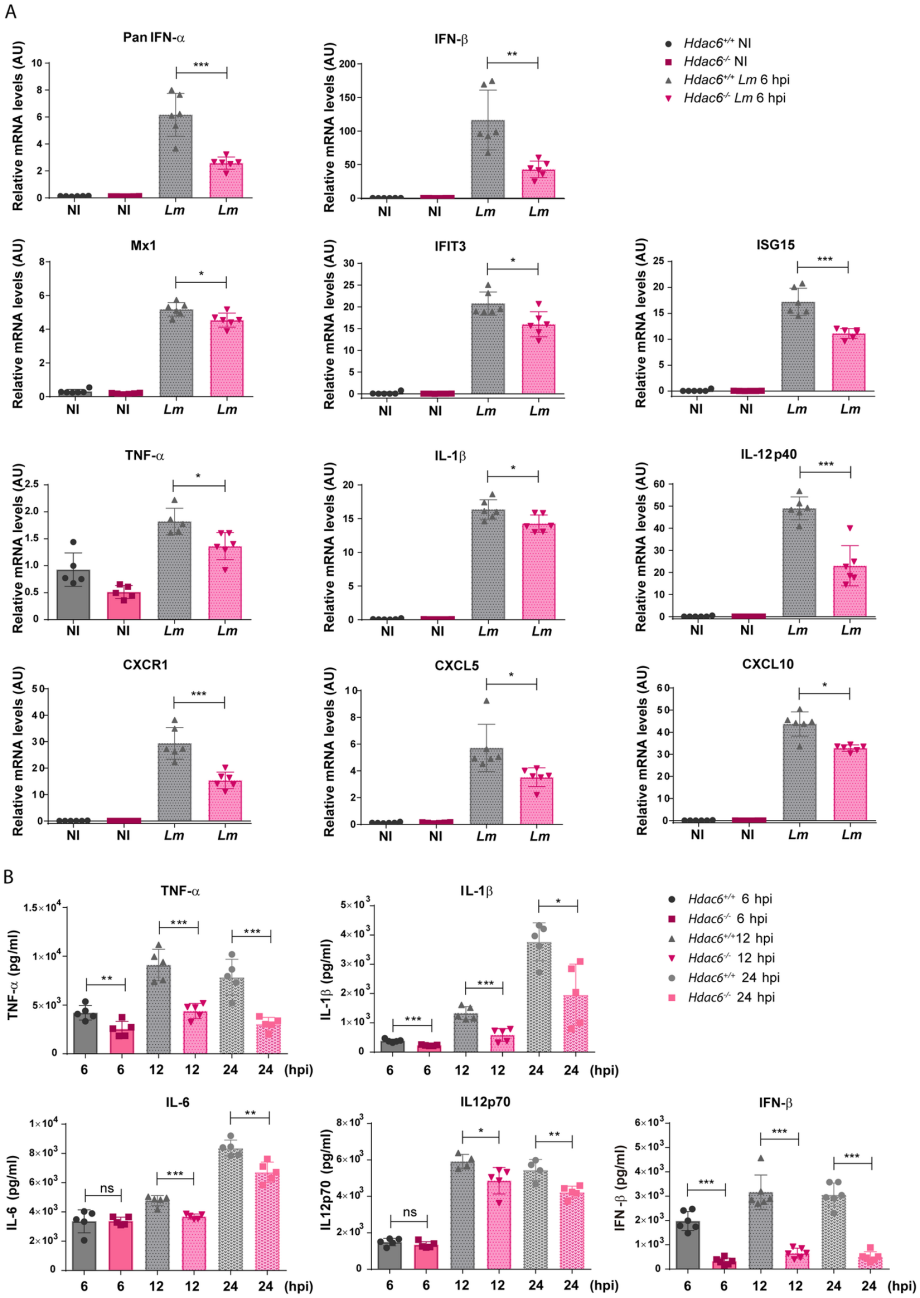
Defective pro-inflammatory cytokine response to *Lm* in *Hdac6*^*-/-*^ BMDCs. A) PCR analysis of type-I interferons (PanIFN-α and IFN-β), interferon downstream proteins (Mx1, IFIT3 and ISG15), pro-inflammatory cytokines (TNF-α, IL-1β and IL-12p40) chemokine receptor (CXCR1) and chemokines (CXCL5 and CXCL10) of *Hdac6*^*+/+*^ and *Hdac6*^*-/-*^ BMDCs non-infected (NI) and infected with *Lm* at 6 hpi (arbitrary units). ***p≤0.001, ** p≤0.01, * p≤0.05; n = 5–6. B) ELISA analysis of the pro-inflammatory cytokines TNFα, IL1β, IL6 and IL12p70 (pg/ml) and IFN-β in supernatants of *Hdac6*^*+/+*^ and *Hdac6*^*-/-*^ BMDCs at 6, 12 and 24 hpi with *Lm*. ***p≤0.001, ** p≤0.01, * p≤0.05 ns>0.05 non-significant; n = 5–6.

The effect of HDAC6 on the response of BMDCs to *Lm* was evaluated by measuring pro-inflammatory cytokine gene induction. The relative mRNA levels of type I interferons (interferons α and β) were lower in *Hdac6*^*-/-*^ BMDCs at 6 hpi (Fig 5A). Accordingly, expression of downstream interferon-response genes such as Mx1, IFIT3, and ISG15 was also lower in *Hdac6*^*-/-*^ BMDCs (Fig 5A). Lack of HDAC6 also decreased the relative mRNA levels of the pro-inflammatory cytokines TNFα, IL-1β and IL12p40, indicating impaired cytokine activation after infection (Fig 5A). Similarly, *Hdac6*^*-/-*^ DCs expressed lower levels than their *Hdac6*^*+/+*^ counterparts of the chemokine receptor CXCR1 and chemokines CXCL5 and CXCL10 (Fig 5A). These data demonstrate that *Hdac6*-deficient DCs have a weakened activation response to *Lm* infection at 6 hpi, which suggests a defect in bacterial clearance, consistent with the increased bacterial load in these cells. To confirm these data, we monitored pro-inflammatory cytokines and IFN-β in the supernatants of *Lm*-infected DCs. Early after infection, TNFα, IL-1β, IL-6, IL12p70 and IFN-β levels were lower in supernatants from *Hdac6*^*-/-*^ cells than in those from *Hdac6*^*+/+*^ cells, and this difference held at 12 and 24 hpi (Fig 5B). To exclude a defect in cytokine secretion, we compared cytokine levels in supernatants (S) with the levels in supernatants plus their corresponding cell pellets (S+P). Both analyses showed decreased cytokine levels in *Hdac6*^*-/-*^ cells, indicating an impaired antibacterial response in *Hdac6*-deficient DCs (S4 Fig).

**Fig 6:**
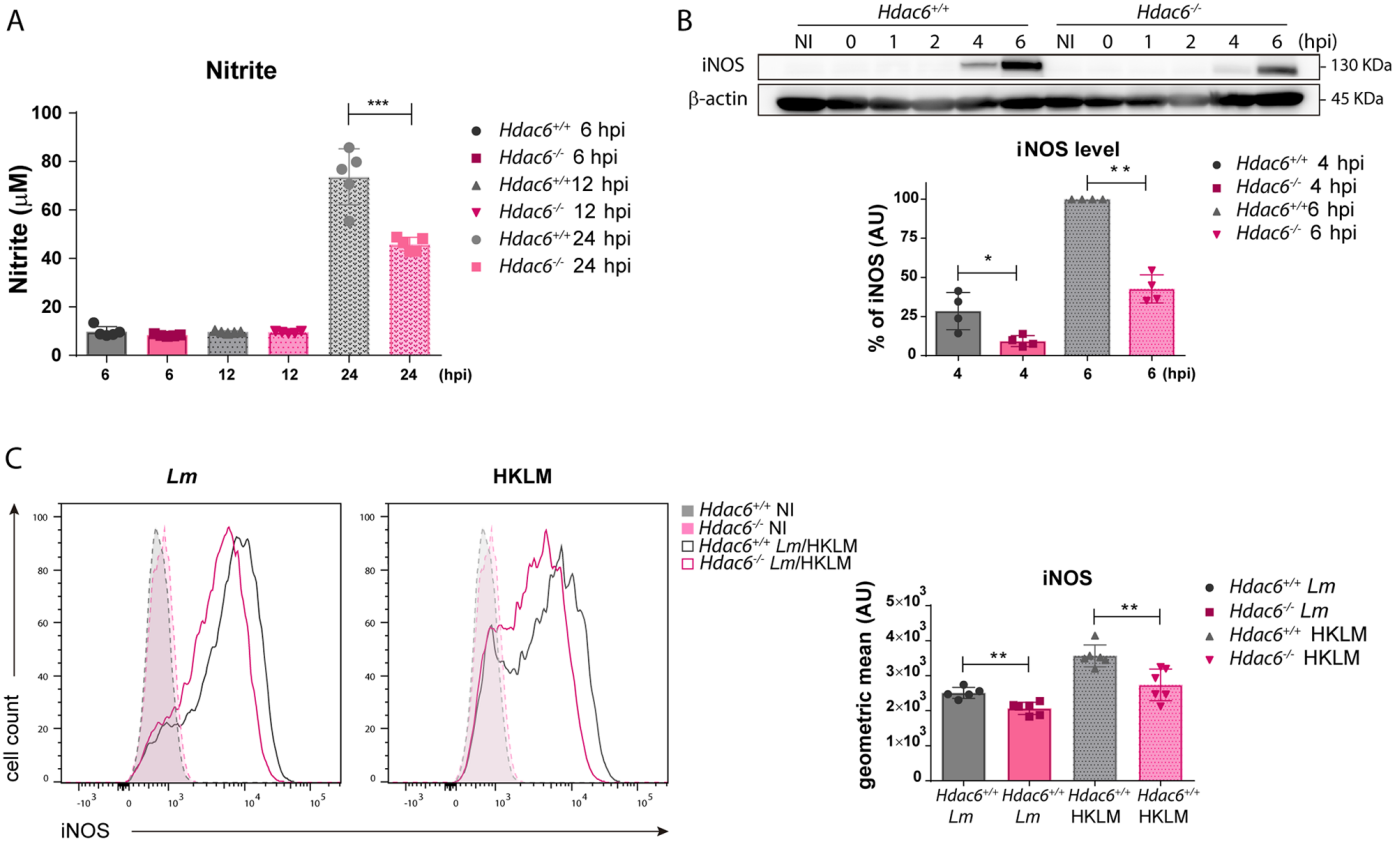
Defective iNOS response to *Lm* in *Hdac6*^*-/-*^ BMDCs. A) *Lm*-activated iNOS activity. Nitrite levels in supernatants of *Lm*-infected BMDCs at 6, 12 and 24 hpi. ***p≤0.001; n = 5. B) Western-blot analysis of iNOS induction over the time-course of *Lm* infection. β-actin was used as a loading control (top panel). The chart shows quantification of iNOS at 4 and 6 hpi. ** p≤0.01, * p≤0.05; n = 4 (lower panel). C) The panel shows representative histograms of iNOS expressed by *Hdac6*^*+/+*^ and *Hdac6*^*-/-*^ BMDCs after exposure to live *Lm* or HKLM for 24 h (left). The right chart shows the geometric mean of iNOS expression. Non-infected (NI) BMDCs were used as a control of iNOS induction. **p≤0.01; n = 6.

Measurement of nitrite in supernatants of infected-BMDCs revealed higher nitric oxide production by *Hdac6*^*+/+*^ DCs than in *Hdac6*^*-/-*^ DCs at 24 hpi (Fig 6A). In agreement, western blot revealed lower levels of inducible nitric oxide synthase (iNOS) in *Hdac6*^*-/-*^ BMDCs at 4 and 6 hpi (Fig 6B), indicating a delay of the enzyme induction in *Hdac6*^*-/-*^ BMDCs. Likewise, flow cytometry after exposure of DCs to live or heat-killed *L*. *monocytogenes* (HKLM) revealed higher expression of iNOS in *Hdac6*^*+/+*^ BMDCs in both cases (Fig 6C). These data support the involvement of HDAC6 in the activation of DC-mediated iNOS microbicidal responses to *Lm* infection and in the clearance of this intracellular pathogen.

### *Hdac6*^*-/-*^ BMDCs show defective activation of Toll-like receptor signalling pathway

**Fig 7:**
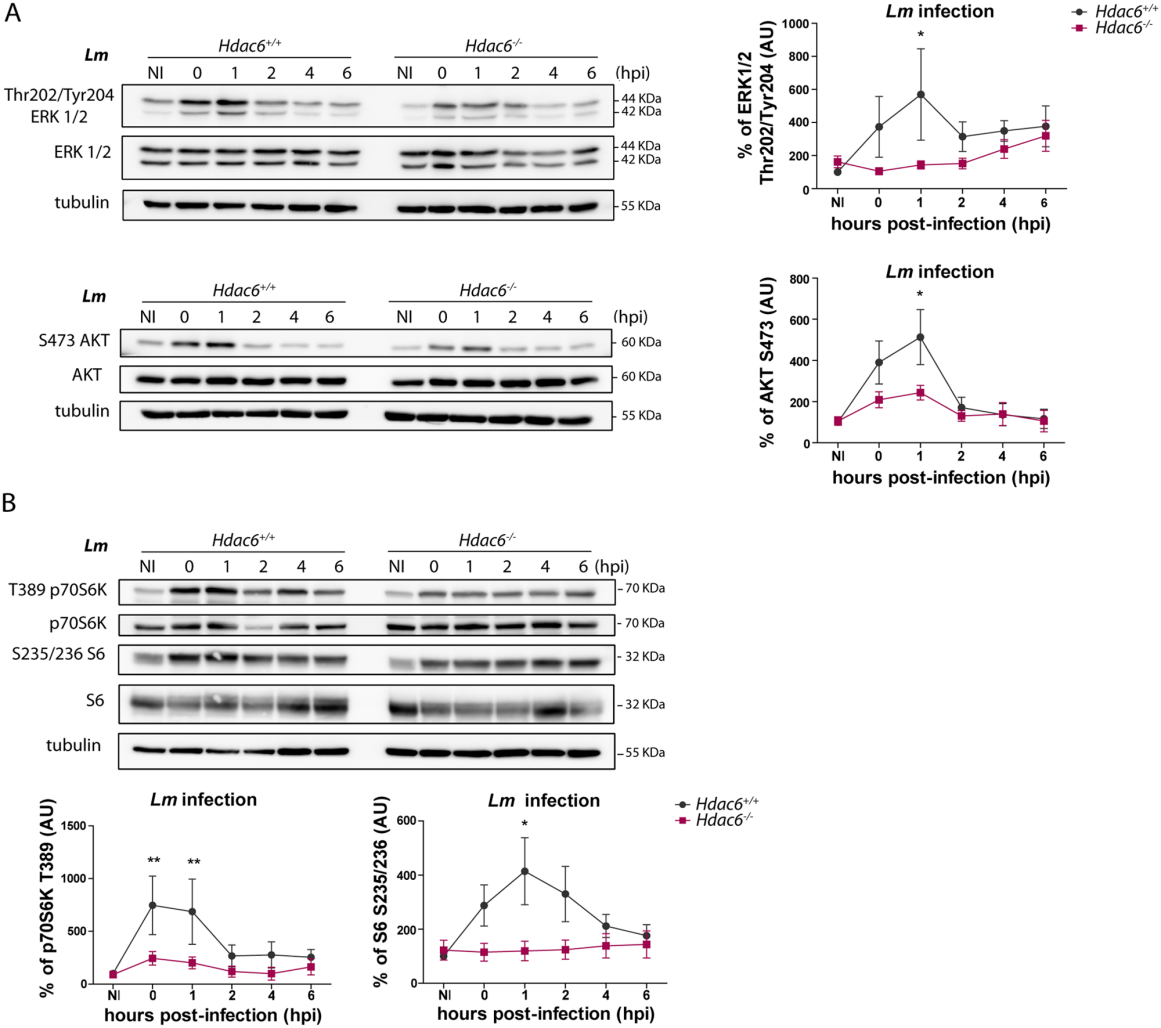
*Hdac6*^*-/-*^ BMDCs show defective activation of the Toll-like receptor signalling pathway. A) Western-blot analysis of MAPK activation over the time-course of *Lm* infection in *Hdac6*^*+/+*^ and *Hdac6*^*-/-*^ BMDCs. Total and phosphorylated ERK and AKT were detected. Tubulin was used as a loading control (left). Accompanying charts show quantification of phERK/totalERK and phAKT/totalAKT ratios relative to the loading control, ns non-significant; n = 7 (right). B) Western-blot analysis of mTORC1 pathway activation over the time-course of *Lm* infection in *Hdac6*^*+/+*^ and *Hdac6*^*-/-*^ BMDCs. Levels of phosphorylated and total p70S6K and S6 were detected. Tubulin was used as a loading control (top panel). Accompanying charts show quantification of php70S6K/total70S6K (n = 5) and phS6/totalS6 (n = 7) ratios relative to the loading control. ** p≤0.01, ns non-significant; (lower panel).

The diminished activation response against *Lm* in *Hdac6*^*-/-*^ BMDCs is consistent with impaired TLR-related signalling. To investigate this question, we determined the phosphorylation levels of TLR downstream mediators by western blot. Compared with *Hdac6*^*+/+*^ BMDCs, *Hdac6*^*-/-*^ BMDCs showed weaker phosphorylation signals for ERK and AKT after *Lm* infection (Fig 7A). We next examined the effect of HDAC6 deficiency on TLR-signalling pathways using other TLR stimuli, including HKLM and LPS. AKT phosphorylation in *Hdac6*^*-/-*^ BMDCs was decreased after LPS or HKLM treatment compared to *Hdac6*^*+/+*^, confirming defective TLR activation (S5 Fig part A). These effects are not related to a defect in *Lm*-induced transcriptional induction since mRNA levels of different *Lm*-related TLRs (TLR1, 2, and 6) were similar in *Hdac6*^*+/+*^ and *Hdac6*^*-/-*^ BMDCs (S5 Fig part B). Moreover, *Hdac6*^*-/-*^ BMDCs showed weaker phosphorylation of mTORC1 pathway proteins (mTORC1 downstream substrates p70S6K and S6), consistent with a less pronounced pro-inflammatory response after TLR-activation by pathogen-associated molecular patterns (PAMPs) (Fig 7B).

**Fig 8:**
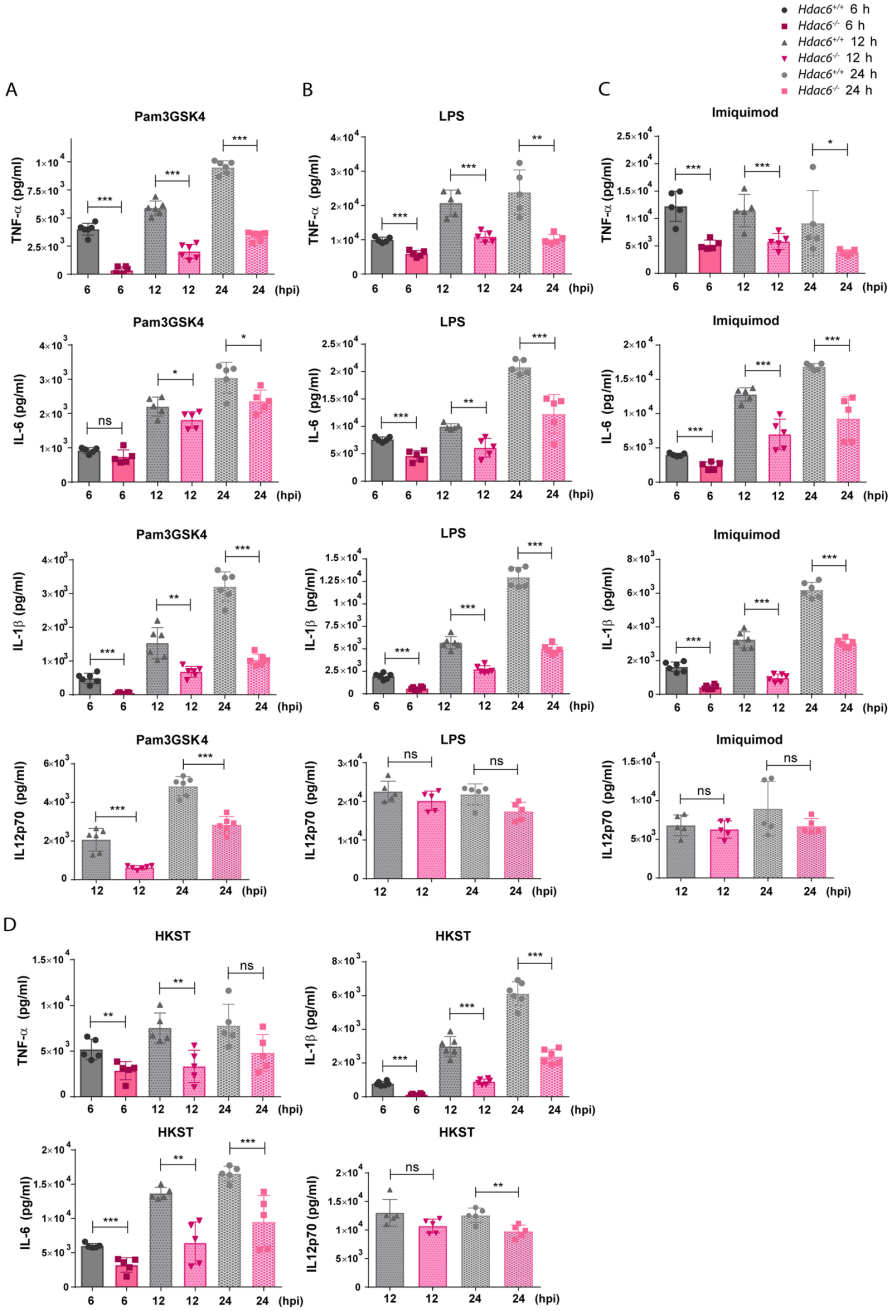
*Hdac6*^*-/-*^ BMDCs show defective inflammatory cytokine response to Toll-like receptor stimuli. A) ELISA analysis of the pro-inflammatory cytokines TNFα, IL-1β, IL-6 and IL12p70 (pg/ml) in supernatants of *Hdac6*^*+/+*^ and *Hdac6*^*-/-*^ BMDCs after treatment for 6, 12 and 24 h with Pam3GSK4. ***p≤0.001, ** p≤0.01, * p≤0.05, ns>0.05 non-significant; n = 5–6. B) ELISA analysis of the pro-inflammatory cytokines TNFα, IL-1β, IL-6 and IL12p70 (pg/ml) in supernatants of *Hdac6*^*+/+*^ and *Hdac6*^*-/-*^ BMDCs after treatment for 6, 12 and 24 h with LPS. ***p≤0.001, ** p≤0.01, ns>0.05 non-significant; n = 5–6. C) ELISA analysis of the pro-inflammatory cytokines TNFα, IL-1β, IL-6 and IL12p70 (pg/ml) in supernatants of *Hdac6*^*+/+*^ and *Hdac6*^*-/-*^ BMDCs after treatment for 6, 12 and 24 h with Imiquimod. ***p≤0.001, * p≤0.05, ns>0.05 non-significant; n = 5–6. D) ELISA analysis of the pro-inflammatory cytokines TNFα, IL-1β, IL-6 and IL12p70 (pg/ml) in supernatants of *Hdac6*^*+/+*^ and *Hdac6*^*-/-*^ BMDCs after treatment for 6, 12 and 24 h with HKST. ***p≤0.001, ** p≤0.01, ns>0.05 non-significant; n = 5–6.

To determine if *Hdac6*^*-/-*^ BMDCs showed a similarly defective response to other TLR agonists, we first examined secretion of pro-inflammatory cytokines in response to agonists of TLR1-2 (Pam3GSK4), TLR-4 (LPS), TLR-7-9 (Imiquimod), and multiple TLRs (heat-killed *Salmonella* Typhimurium; HKST). *Hdac6*^*-/-*^ BMDCs showed a defective cytokine response to these stimuli, determined from the release of TNFα, IL-6, IL-1β and IL12p70 (Fig 8A–8D).

**Fig 9:**
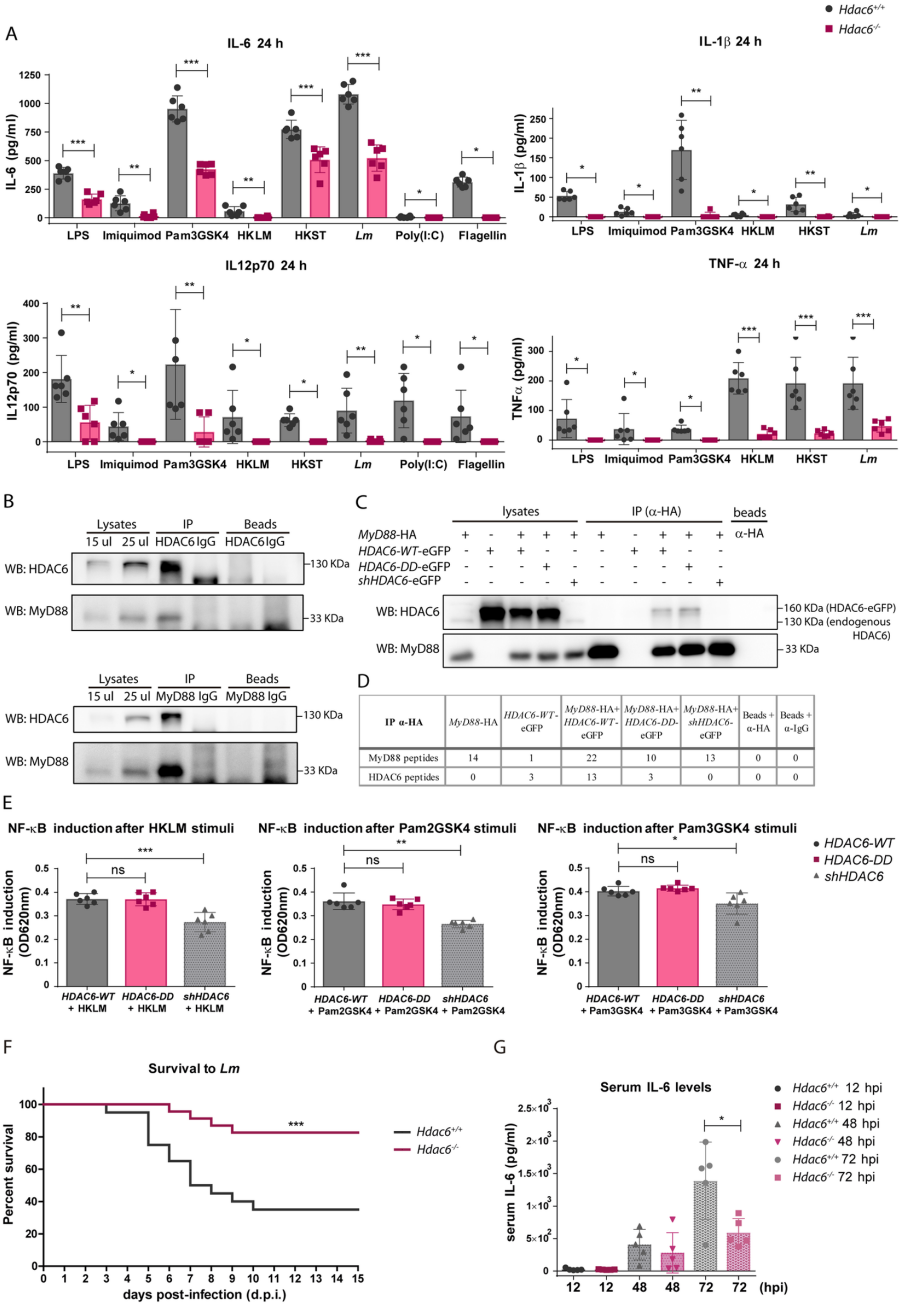
Association of HDAC6 with TLR-adaptor MyD88 and its contribution to the inflammatory response to *Lm*. A) ELISA analysis of the pro-inflammatory cytokines TNFα, IL-1β, IL-6 and IL12p70 (pg/ml) in supernatants of *Hdac6*^*+/+*^ and *Hdac6*^*-/-*^ FLT3L-DCs activated with LPS, Imiquimod, Pam3GSK4, HKLM, HKST, *Lm*, Poly(I:C) and flagellin for 24 h. ***p≤0.001, ** p≤0.01, * p≤0.05; n = 6. B) Immunoprecipitation of endogenous HDAC6 and MyD88 followed by western-blot for both proteins. Immunoprecipitations were carried out using human moDCs after 30 min of stimulation with Pam2GSK4, Pam3GSK4 and HKLM. Endogenous HDAC6 (130 KDa) and MyD88 (33 KDa) are indicated at right of western-blot. Similar results were obtained in two independent experiments. C) Immunoprecipitation of HA (MyD88) followed by western-blot for HDAC6 and MyD88. Immunoprecipitations were carried out using different HDAC6-eGFP plasmids co-transfected with MyD88-HA in HEK-Blue hTLR2 cell line after 30 min of stimulation with HKLM. Over-expressed (HDAC6-eGFP, 160 KDa) and endogenous HDAC6 (130 KDa) are indicated at right of western-blot. Similar results were obtained in four independent experiments. D) Immunoprecipitation of HA (MyD88) followed by mass spectrometry analysis. Immunoprecipitations were carried out using different HDAC6-eGFP plasmids co-transfected with MyD88-HA in HEK-Blue hTLR2 cell line after 30 min of stimulation with HKLM. The number of unique MyD88 and HDAC6 peptides is indicated. No acetylated peptides from MyD88 were detected in any sample. Similar results were obtained in four independent experiments. E) Graph of NF-κB induction in transfected *HDAC6-WT*, *HDAC6-DD* and *shHDAC6* HEK-Blue hTLR2 cell line after activation with HKLM, Pam2GSK4 and Pam3GSK4 during 8 h. NF-κB induction was calculated by the ratio of the signal of stimulated cells with its corresponding transfected cells in basal condition (without stimuli), ***p≤0.001, ** p≤0.01, * p≤0.05, ns>0.05 non-significant; n = 6. F) Survival curve to intravenous injection with a lethal dose of *Lm* in *Hdac6*^*+/+*^ and *Hdac6*^*-/-*^ is showed. This curve corresponds to two different experiment of survival to *Lm* with a n = 24–21. ***p≤0.001. G) Pro-inflammatory cytokine IL-6 was measured in sera of *Hdac6*^*+/+*^ and *Hdac6*^*-/-*^ mice intravenously injected with a lethal dose of *Lm* at 12, 48 and 72 hpi. *p≤0.05, n = 5.

To assess the pro-inflammatory cytokine response to TLR3 and TLR5 ligands, we generated Fms-related tyrosine kinase 3 ligand dendritic cells (FLT3L-DCs). Differentiation with the cytokine FLT3L yielded similar percentages of CD24^+^ and CD24^-^ subpopulations (CD11c^+^CD11b^+^B220^-^CD24^+^ and CD11c^+^CD11b^+^B220^-^CD24^-^, respectively) from *Hdac6*^*+/+*^ and *Hdac6*^*-/-*^ DCs, indicating that differentiation is unaffected by HDAC6 absence (S6 Fig part A). The TLR agonists Pam3GSK4 (TLR1-2), Poly(I:C) (TLR3), LPS (TLR4), flagellin (TLR5), Imiquimod (TLR-7-9), *Lm*, HKLM, and HKST (which activates several TLRs simultaneously) elicited similar cytokine secretion profiles in GM-CSF DCs and FLT3L-DCs (Fig 9A and S6 Fig part B compared to Fig 8A–8D). *Hdac6*^*-/-*^ DCs of both derivations showed an impaired cytokine response to each TLR agonist, indicating that HDAC6 likely regulates a common TLR signalling adaptor.

In this view, the TLR adaptor MyD88 participates in the transmission of signals by all TLRs except for TLR3. We decided to study MyD88 levels in a time-course infection with *Lm* by western blot, demonstrating that the quantity of this molecule was the same between *Hdac6*^*+/+*^ and *Hdac6*^*-/-*^ DCs and remaining stable during infection (S6 Fig part C). Remarkably, MyD88-HDAC6 molecular association was observed by co-immunoprecipitations of endogenous proteins using human dendritic cells after Pam2GSK4, Pam3GSK4 and HKLM stimulation (Fig 9B). Likewise, the MyD88 immunoprecipitation in *MyD88*- and *HDAC6*-overexpressed HEK cell line was also able to co-precipitate HDAC6 (S6 Fig part D). These results were corroborated by mass spectrometry analysis of MyD88 immunoprecipitates; which in addition detected two acetylated peptides corresponding to MyD88 (S6 Fig parts E and F). The same approach was used to determine MyD88-HDAC6 molecular association after TLR-2 activation with HKLM using a constitutively expressed human TLR-2 HEK cell line, rendering the same result (Fig 9C). This association is also maintained using a double-deacetylase domain mutant of HDAC6 (H216A:H611A), called HDAC6-DD, indicating that HDAC6-MyD88 interaction is independent of its catalytic activity (Fig 9C and S6 Fig part D). The knock-down of HDAC6 expression using a small harping plasmid (sh-HDAC6) blocked this interaction (Fig 9C and S6 Fig part D). However, no acetylated peptides could be detected in the mass spectrometry analysis of the MyD88 immunoprecipitation from HKLM-stimulated TLR-2 HEK cell line (Fig 9D). Moreover, assessment of NF-κB induction in TLR-2-expressing HEK cell line after HKLM, Pam2GSK4 and Pam3GSK4 stimulation shows lower activation only in shHDAC6 transfected cells, without affecting the activity of HDAC6-DD-transfected ones (Fig 9E).

Taking into account all these data, HDAC6 associates with the TLR-adaptor molecule MyD88, and the absence of HDAC6 in DCs seems to diminish the TLR-response after a variety of stimuli, underlining the scaffold role of HDAC6 in determining the ability of MyD88 to mediate TLR signalling.

To ascertain the defective TLR-dependent inflammatory response *in vivo* due to the molecular association of MyD88-HDAC6, *Hdac6*^*+/+*^ and *Hdac6*^*-/-*^ mice were intravenously injected with a lethal dose of *Lm* (Fig 9F) [44]. A protective effect against *Lm*-induced septic shock was observed from 3 to 10 days post-infection (dpi) (Fig 9F). Accordingly, lower levels of the pro-inflammatory cytokine IL-6 were detected in the serum of *Hdac6*^*-/-*^ mice at 72 hpi, highlighting a reduced systemic cytokine-driven inflammatory response after *Lm* infection in these mice (Fig 9G).

## Discussion

**Fig 10:**
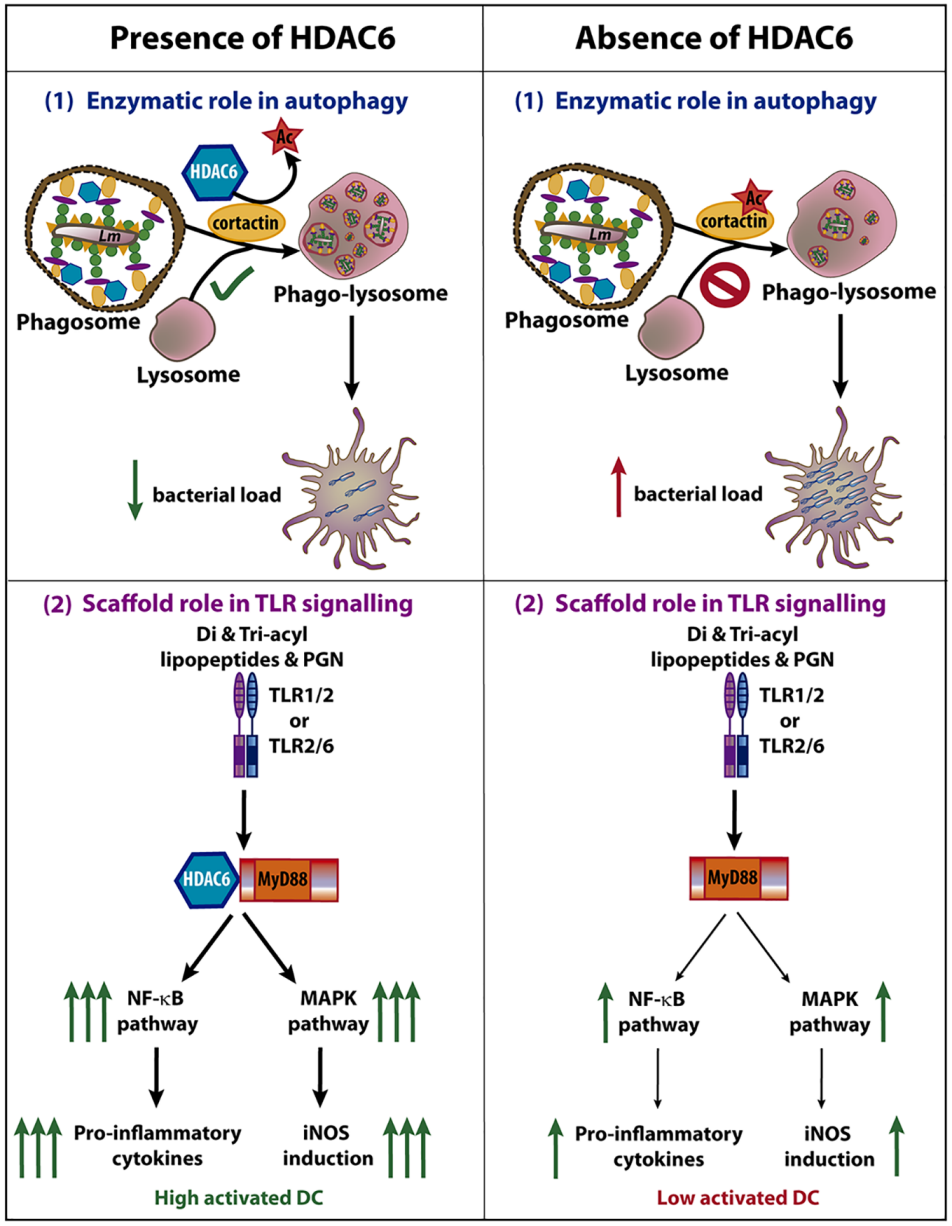
Dual role of HDAC6 during *Lm* infection in dendritic cells. The scheme shows the involvement of HDAC6 in two different functions of dendritic cell during *Lm* infection, the autophagy and the TLR signalling. **(1)** The fusion of phagosome with lysosome is dependent on cortactin and F-actin. The deacetylation of cortactin by HDAC6 allows the correct fusion, followed by an autophagic clearance of *Lm*. The absence of this enzyme delays the fusion of phagosome and lysosome, facilitating *Lm* to escape from phagosome leading to an increased bacterial load. **(2)** Di- and tri-acyl lipopeptides and peptidoglycan (PGN) of *Lm* are recognized by TLR1/2 or TLR2/6, activating the TLR pathway. HDAC6 is able to interact with the TLR-adaptor protein MyD88 which caused an enhanced down-stream signalling of TLR pathway, increasing NF-κB and MAPK activation. This stronger activation (independent on HDAC6 enzymatic activity) results in higher pro-inflammatory cytokine production and iNOS induction, reinforcing the ability of the DC to combat against this intracellular pathogen. Although the absence of HDAC6 does not fully abolish the activation of the DC, a lower induction of NF-κB and MAPK pathways promotes a lower activation of the anti-bacterial transcriptional program of the DCs. Note that both processes occur during *Lm* infection and the pro-inflammatory cytokines and iNOS induction can impact on the autophagic process. The images in the figure are not scaled.

Recent studies have revealed the involvement of HDAC6 in the innate immune response against Influenza Virus A (IVA), Sendai virus (SeV), and vesicular stomatitis virus (VSV) [21–24]. Given the similarities between the innate responses to viruses and intracellular bacteria, this prompted us to investigate the role of HDAC6 in a model of *Lm* infection. In this work, we demonstrated a dual role of HDAC6 in the innate response against *Lm*, not only due to its enzymatic activity but also dependent of its function as a scaffold (Fig 10). Our data clearly demonstrate that *Hdac6*^*-/-*^ BMDCs have an impaired immune response against *Lm* and *S*. Typhimurium infection *in vitro*. Moreover, higher *Lm* titres observed in *Hdac6*^*-/-*^ dendritic cells, M-CSF-derived macrophages and peritoneal macrophages were corroborated during *in vivo Lm* infection at 6 hpi in various myeloid subsets of the spleen. The absence of this effect during BMDC *in vitro* infection by the non-intracellular bacteria *S*. *aureus* and *E*. *coli* DH5α indicates that *Hdac6*^*-/-*^ BMDCs are specifically unable to efficiently clear intracellular pathogens. HDAC6 is involved in two of the most important cellular clearance systems, autophagy and ubiquitin-proteasome system (UPS) [9, 13]. In the case of *Lm* and *S*. Typhimurium, the main molecular mechanism for degradation of vesicle-contained bacteria is fusion with lysosomes in a process called autophagy [45–47]. In agreement with this, our data show that impaired phagosome-lysosome fusion underlies the phenotype observed in *Hdac6*^*-/-*^ BMDCs. Unsuccessful fusion depends on acetylated-cortactin in *Hdac6*-deficient cells [9]. A similar mechanism has been described in *Hdac6*-deficient MEFs during quality-control autophagy [9]. We demonstrated that in *Hdac6*^*-/-*^ BMDCs co-localized higher levels of acetylated-cortactin with intracellular *Lm*. The delay in vesicle fusion caused by the acetylation of cortactin, impairs the phagosome-lysosome fusion and provides more opportunities for phagosome-containing bacteria to escape to the cytosol, resulting in the higher bacterial load detected in *Hdac6*^*-/-*^ BMDCs. Based on this experimental evidence, it is conceivable to postulate that the enzymatic activity of HDAC6 on its substrate cortactin controls autophagy of intracellular bacteria for their efficient clearance (Fig 10).

Pharmacological autophagy inhibitors erased the observed differences between *Hdac6*^*+/+*^ and *Hdac6*^*-/-*^ BMDCs. Conversely, rapamycin did not overcome the *Hdac6*^*-/-*^ autophagy defect, indicating a defect in phagosome-lysosome fusion. However, other authors have reported opposite observations using pan-HDAC inhibitors or specific inhibitors of HDAC6 during infection of human macrophages with the *Gram*-negative intracellular pathogens *S*. Typhimurium and *E*. *coli* [48]. These inhibitors, when added at the time of infection, increase mitochondrial ROS production [48]. However, overnight pre-treatment before infection hampered bacterial clearance and reduced phagocytosis [48]. These data indicate that specific HDAC6 chemical inhibitors can have side-effects, including effects on other HDAC members, potentially interfering with the acetylation of other substrates upstream of cortactin that also have a role during bacterial infection. Our genetic approach unequivocally assigns a specific role to HDAC6 in innate cells during bacterial infection.

Although we observed an impairment in phagosome-lysosome fusion, we cannot rule out an involvement of HDAC6 in the anti-microbial response through its BUZ domain, with a contribution from ubiquitin. The characterized interaction between p62 and HDAC6 through their ubiquitin-binding domains provides a clue about the possible role of ubiquitin in the activation of innate immunity through the recognition of ubiquitinated-molecules [15]. For example, the ubiquitin-binding regions of HDAC6 and p62 are both essential for MyD88 aggregation and the downstream activation of MyD88-dependent signal transduction [49]. Furthermore, ubiquitin-binding platforms formed by HDAC6 and p62 are able to interact with interferon stimulated gene 15 (ISG15) to eliminate ISGylated proteins tagged after interferon stimulation by autophagy [50]. HDAC6 is able to bind to either mono- and poly-ubiquitinated proteins, but shows a preference for proteins modified with k63-linked ubiquitin chains, which share structural similarities with ISG15 [51]. *S*. Typhimurium is decorated with this kind of ubiquitin chain for recognition by host cells, and can be recovered with phagosome proteins to initiate autophagy [52, 53]. Nevertheless, our data demonstrate that autophagy induction does not differ between *Hdac6*^*+/+*^ and *Hdac6*^*-/-*^ BMDCs, indicating that this phenotype is due to p62 accumulation in *Hdac6*^*-/-*^ BMDCs as a consequence of impaired phagosome-lysosome fusion. Intact autophagy activation in *Hdac6*^*-/-*^ BMDCs could be explained by compensatory p62 binding to ubiquitinated bacteria in the absence of HDAC6.

Our data also underscore other different function of HDAC6, independent of its enzymatic activity, in innate immune response to intracellular bacteria and various TLR stimuli (Fig 10). Hence, we provide evidence for a dampened inflammatory response in the absence of HDAC6, as shown by lower RNA levels of pro-inflammatory cytokines, chemokines, type-I interferons, and interferon-related proteins in *Hdac6*^*-/-*^ BMDCs than in *Hdac6*^*+/+*^ cells at 6 hpi, as well as the lower pro-inflammatory cytokine production and IFN-β secretion by infected *Hdac6*^*-/-*^ BMDCs from 6 to 24 hpi. Moreover, *Hdac6*^*-/-*^ BMDCs showed diminished iNOS induction at 6 hpi associated with low nitrite production and iNOS expression at longer times of *Lm* infection (24 hpi). These results agree with a lower phosphorylation of the MAPK pathway after *Lm* infection in *Hdac6*^*-/-*^ dendritic cells, controlling the activation of AP-1 family transcription factors, which is necessary to switch inflammatory responses on [54]. In addition, the lower phosphorylation of mTORC1 pathway components in *Hdac6*^*-/-*^ DCs is consistent with a lower pro-inflammatory response, as reported in trained macrophages and dendritic cells, in which a metabolic switch to glycolysis has been described [55]. These data may indicate that HDAC6 also appears to play a role in the activation of mTOR pathway after *Lm* infection to initiate the antibacterial transcriptional response to combat this pathogen. In summary, our results reveal a defect in DC activation after *Lm* entry.

Remarkably, this impaired anti-inflammatory response in *Hdac6*^*-/-*^ BMDCs was also observed with other TLR-agonists such as LPS, Imiquimod, poly(I:C) and Pam3GSK4, highlighting HDAC6 as an important player in TLR signalling activation. Broad-spectrum HDAC inhibitors such as TSA exert immunosuppressive effects [56]. Genome-wide expression profiling arrays have revealed that 60% of genes transcriptionally increased by TLR2 or TLR4 stimulation are inhibited in TSA-treated cells, whereas 16% of genes are potentiated [56]. However, these observations do not provide any demonstrative evidence for a specific role of HDAC6, since other HDACs may also be involved.

Because GM-CSF-derived DCs express low levels of TLR3 and 5 in the membrane, we stimulated FLT3-L-derived DCs with poly(I:C) and flagellin to measure pro-inflammatory cytokines [57, 58], noting maintained failure in the inflammatory response in *Hdac6*^*-/-*^ cells. Moreover, we detected impaired responses to PAMPs activation in GM-CSF-derived and FLT3L-derived *Hdac6*^*-/-*^ DCs. In addition, all TLRs except for TLR3 signal through the adaptor MyD88, and the result obtained with the TLR3 ligand poly(I:C) was similar to that showed for the rest of TLR stimuli, thereby indicating that the TLR3-response is also affected by absence of HDAC6. In this regard, these data are in agreement with a recent study showing that acetylated retinoic-acid-inducible gene-1 (RIG-1) makes *Hdac6*^*-/-*^ cells less sensitive to the presence of RNA viruses, resulting in a higher viral titre [59]. While this mechanism could explain the difference between the response of *Hdac6*^*+/+*^ and *Hdac6*^*-/-*^ FLT3L-DCs to TLR3 stimulation, the deficient activation by other TLRs in *Hdac6*^*-/-*^ DCs also requires an explanation. In this respect, we demonstrate the molecular association of HDAC6 with MyD88 with endogenous proteins and in an overexpression system. Conceivably, the depletion of HDAC6 and therefore prevention of HDAC6-MyD88 binding, could inhibit TLR-2-signalling pathway activation, which is in accordance with a lower NF-κB induction measured in *Hdac6*^*-/-*^ cells after various TLR-2 agonist stimulation. A diminished NF-κB induction in *Hdac6*^*-/-*^ cells could explain a reduced initiation of the pro-inflammatory response observed in *Hdac6*^*-/-*^ dendritic cells, needed to alert the innate and adaptive immune response to *Lm*. However, NF-κB activity of HDAC6-DD-transfected HEK cell line after TLR-2 stimuli did not display any significant change compared to HDAC6-WT-transfected ones, supporting that enzymatic activity of HDAC6 is not involved in this signalling pathway. Two acetylated peptides corresponding to MyD88 have been found in basal condition in HEK transfected with HDAC6-DD and shHDAC6 constructions, which are different from the residue previously described in MyD88 [60]. However, no changes in the acetylation marks on MyD88 were detected after HKLM incubation, highlighting the scaffold role of HDAC6 in the proper activation of TLR-signalling pathway (Fig 10). Unexpectedly, a protective effect against systemic infection to a lethal dose of *Lm* were observed in *Hdac6*^*-/-*^ mice. The reduced level of the inflammatory cytokine IL-6 detected in *Hdac6*^*-/-*^ mice are in accordance with its higher resistance to *Lm* infection. The defective systemic inflammatory response after *Lm* infection of *Hdac6*^*-/-*^ mice may indicate an impaired TLR-response in the absence of HDAC6 and might therefore be attributed to the absence of the molecular association of MyD88 and HDAC6. In this regard, mice resistance to *Lm* infection can be mediated by sequential MyD88-independent and -dependent responses [61–64]. However, the role of TLR-2 during *Lm* infection does not appear to be clear enough [62, 63]. On one hand, *Tlr-2*^*-/-*^ mice display a deficit in circulating TNF-α and IL-12p40 production during intravenously injected *Lm* infection combined with a lower mice survival and increased bacterial burden in the liver [61, 63]. Other authors have obtained similar resistance to intraperitoneal-injected *Lm* infection between *Tlr-2*^*-/-*^ and *Tlr-2*^*+/+*^ mice, indicating that different inoculation routes of bacteria may render different immune outcomes [62]. Although handling and direct killing of *Lm* by activated macrophages can be mediated by TLR-2- and MyD88-independent mechanisms, the role of TLR-signalling has been observed necessary for nitric oxide and cytokine production [61, 63]. In fact, MyD88 not only works as TLR-adaptor protein, but also as adaptor of IL-1 and IL-18 receptors, both cytokine responses affected in *Lm*-MyD88^-/-^ mice [62, 63, 65].

Overall, our data support a dual role for HDAC6 in the regulation of innate immunity against intracellular bacteria. An increased bacterial load in different *Hdac6*^*-/-*^ myeloid cells can be explained by the autophagy mechanism, where a permanently acetylated cortactin may impair the phagosome-lysosome fusion, necessary for the clearance of this pathogen. Our experiments also show the importance of HDAC6 in DC activation, uncovering a novel mechanism of HDAC6 action mediated by the appropriate signalling via the TLR pathway, due to the association of HDAC6 with the TLR-adaptor protein MyD88. This molecular association seems to be required for a response to TLR stimuli to initiate the inflammatory response of an activated dendritic cell. Taken together, both HDAC6 functions described in this manuscript, reinforce the importance of this molecule to combat intracellular bacteria as *Lm* by autophagy and to completely activate the inflammatory response after TLR activation.

## Materials and methods

### Ethical statement

Mice were housed under specific pathogen-free conditions at the Centro Nacional de Investigaciones Cardiovasculares Carlos III (CNIC), and experiments were approved by the CNIC Ethical Committee for Animal Welfare and by the Spanish Ministry of Agriculture, Food, and the Environment. Animal care and animal procedures license were reviewed and approved by the local Ethics Committee for Basic research at the CNIC Ethical Committee for Animal Welfare and the Órgano Encargado del Bienestar Animal (OEBA) del Gabinete Veterinario de la Universidad Autónoma de Madrid (UAM). This committee approved the document with an associated identification number PROEX 158/15 (CNIC 04/15).

Buffy coats of healthy donors were received from the Blood Transfusion Center of Comunidad de Madrid, and all donors signed their consent for the use of samples for research purposes. All the procedures using primary human cells were approved by the Ethics Committee of the Hospital Universitario de la Princesa.

### Mice

*HDAC6*^-/-^ mice were generated through targeting of exons from 10 to 13 by inserting a neomycin (Neo) and zeocin (Zeo) cassette, resulting in the disruption of the first catalytic domain of HDAC6 [66]. These mice were intercrossed on a C57BL/6 background to generate sex and age matched wild-type (*wt*) and knockout.

### Bacteria strains

We used the *Listeria monocytogenes EGD* (BUG 600) strain, provided by Dr. Esteban Veiga (Centro Nacional de Biotecnología, CNB, Madrid). *Staphylococcus aureus* 132 and *Escherichia coli* K12, strain DH5α, were purchased from Invitrogen. BUG600 and *S*. *aureus* bacteria were grown in BHI broth. RFP-expressing *Listeria monocytogenes* (RFP-*Lm*) was provided by Dr Carlos Ardavín´s laboratory (Centro Nacional de Biotecnología, CNB, Madrid). *Salmonella enterica* serovar Thyphimurium strain SL1344 was provided by Dr. J. Garaude (Centro Nacional de Investigaciones Cardiovasculares, CNIC, Madrid). SL1344 and DH5α bacteria were grown in LB broth supplemented with 50 μg/ml streptomycin (Sigma). For phagocytosis experiments, *Lm* and *S*. *aureus* were grown overnight in Brain Herat Infusion (BHI) broth and *E*. *coli* and *S*. Thyphimurium in Luria-Bertani (LB) broth with shaking, diluted 1/50, and grown until log-phase (optical density 0.8–1.2 at 600 nm) without shaking. Bacteria were washed with phosphate-buffered saline (PBS) to remove LB salts before addition to cells.

### Cell culture

The HEK293T cell line (ATCC) was cultured in DMEM medium (Sigma) containing 10% FBS (Invitrogen), 2 mM L-glutamine, 100 mg/ml penicillin and 100 mg/ml streptomycin. HEK Blue hTLR2 cell line (Invivogen), the HEK293 cell line expressing human TLR2, CD14 and NF-κB-SEAP (secreted embryonic alkaline phosphatase) reporter gene was cultured in DMEM medium (Sigma) containing 10% FBS (Invitrogen), 2 mM L-glutamine, 100 μg/ml Normocin (Invivogen) and 1X HEK-Blue Selection (Invitrogen).

### Generation of bone marrow-derived dendritic cells (GM-CSF) and macrophages (M-CSF)

Mouse primary dendritic cells (BMDCs) and macrophages (BMDMs) were obtained from bone marrow cell suspensions after culture on non-treated 150-mm Petri dishes in complete RPMI 1640 supplemented with 10% FBS, 2 mM L-glutamine, 100 mg/ml penicillin, 100 mg/ml streptomycin, 50 mM 2-ME, and 20 ng/ml granulocyte-macrophage colony-stimulating factor (GM-CSF, PeproTech, London, U.K.) for BMDCs and macrophage colony-stimulating factor (30% mycoplasma-free L929 cell supernatant, NCBI Biosample accession number SAMN00155972) for BMDMs. BMDCs were collected at day 9 and BMDCs were characterized as CD11c^+^MHC-II^+^Gr-1^-^ cells by flow cytometry. BMDMs were collected at day 6 and BMDMs were characterized as CD11b^+^F4/80^+^ or CD11b^+^CD64^+^ cells.

### Generation of bone marrow-derived dendritic cells (FLT3L)

Bone marrow cell suspensions were culture on treated 6 well plates in complete RPMI 1640 supplemented with 10% FBS, 2 mM L-glutamine, 100 mg/ml penicillin, 100 mg/ml streptomycin, 50 mM 2-ME, and 150 ng/ml (FLT3L, PeproTech, London, U.K.). After 9–11 days of differentiation cells were collected to be characterized by flow cytometry as CD11c^+^B220^-^CD11b^+^CD24^-^ (60% of the culture) and CD11c^+^B220^-^CD11b^+^CD24^+^(40%).

### Obtainment of thioglycollate-elicited macrophages (TEMs)

Mice received peritoneal injections with 1ml 4% TG. The peritoneal exudate was collected after 4 days and cultured in complete RPMI 1640 supplemented with 10% FBS, 2 mM L-glutamine, 100 mg/ml penicillin, 100 mg/ml streptomycin, and 50 mM 2-ME. To enrich the culture for macrophages, non-adherent cells were eliminated after a few hours by washing five times with warm PBS and gentle swirling.

### Obtainment of human monocyte-derived dendritic cells (moDCs)

Peripheral blood mononuclear cells (PBMCs) from Buffy coats of healthy donors were isolated using Biocoll separating solution (Millipore) by centrifugation at 700 g 30 min at RT. Monocytes were purified from peripheral blood mononuclear cells (PBMCs) by an adherence step at 37°C in incomplete RPMI 1640 medium during 1 h. Non-adherent cells were removed and adherent monocytes were washed three times with warm 1xPBS to remove residual PBMCs. Monocytes were cultured in complete RPMI 1640 supplemented with 10% FBS, 2 mM L-glutamine, 100 mg/ml penicillin, 100 mg/ml streptomycin, 500 U/ml IL-4 (R&D) and 500 U/ml GM-CSF (Immunotools) for 6 days. Fresh medium and cytokines were added every 48 hours to differentiate monocytes to immature human dendritic cells. Cells were characterized by flow cytometry as HLA-DR^+^CD3^-^DC-SIGN^+^CD14^-^CD11c^+^. Activation of dendritic cells was induced with Pam2GSK4, Pam3GSK4 and HKLM for 30 min (Invivogen).

### *In vitro Lm*-infection of BMDCs, BMDMs and TEMs

Cells were incubated with *Lm* and assessed for survival to gentamicin exposure [67]. Cells were infected with *Lm* at a multiplicity of infection (MOI) of 10 for 30 min at 37°C. To determine the number of bacteria entering the cells, extracellular bacteria were killed by treatment with 100 μg/ml gentamicin (Sigma-Aldrich, St. Louis, MO) for an additional 30 min at 37°C. Then, infected cells were washed with PBS three times and lysed with 0.05% Triton X-100 (Sigma-Aldrich, St. Louis, MO) in distilled water. Serial dilutions were seeded on brain-heart infusion (BHI) agar plates and CFUs were counted after 36 hours.

### *In vivo Lm* systemic infections

*Hdac6*^*+/+*^ and *Hdac6*^*-/-*^ were intravenously injected with *Listeria monocytogenes* EGD (125.000 CFUs/mouse) using a 29-gauge needle. For survival experiments mice were monitored twice a day in order to detect casualties during 15 days of infection.

### Determination of CFUs in target organs

After 12, 24, 48 and 72 hpi, mice were perfused with 1X PBS to clean blood form organs and spleens and livers were weight. To determine bacterial load, spleens and livers were digested with 0.1 mg/ml type IV collagenase and 0.5 mg/ml DNAse I (Roche, Mannheim, Germany) for 30 min at 37°C. After digestion, organs were homogenized in 70 μm filters and red blood cells were lysed with ammonium chloride potassium lysis buffer (ACK, Sigma). Splenic cell suspensions were resuspended in PBS and cells were counted. Serial dilutions were grown on BHI agar plates. CFUs were counted after 36 hours of incubation at 37°C. CFUs were calculated by cell number and by gram per organ.

### Antibodies and reagents

Antibodies were used in western blotting, flow cytometry and immunofluorescence; detailed information is available in S1 Table. Poly-L-lysine (PLL) was purchased from Sigma. Phalloidin-Alexa488 and 647 were from BD Biosciences. Zenon Alexa Fluor 488 rabbit IgG labelling kit, DAPI and Prolong Gold anti-fade mounting medium were from Thermofisher Scientific. Anti-human CD3 antibody (T3b hybridoma) was generated in Dr. F. Sánchez-Madrid laboratory (Hospital Universitario de la Princesa, HUP, Madrid) [68]. Rapamycin, bafilomycin A1, 3-MA, cloroquine, NH_4_Cl, 1400W and DPI were from Sigma-Aldrich.

### Gene overexpression and silencing

HEK293 cells were co-transfected with plasmids encoding human MyD88 fused to the HA-tag (Addgene plasmid #12287) together with plasmids encoding *HDAC6-WT* or double deacetylase domain mutant DD (mutated human HDAC6-H216A/H611A) fused to the eGFP tag (*HDAC6-WT* and *HDAC6-DD* have been previously described [26]. When indicated, cells were co-transfected with the appropriate small harping RNA plasmid pLVX-IRES-ZsGreen1, where *shHDAC6*-2049 (TRCN0000004842) was cloned between BamH1 and EcoR1 sites. Cells were transfected using Lipofectamine 2000 (Invitrogen). Experiments were performed after 24 h after transfection.

### RNA extraction and real-time quantitative PCR

RNA from mouse BMDCs was isolated with the QIAGEN RNeasy Kit (Qiagen). Residual DNA contamination was removed with the Turbo DNA-free Kit (Ambion). Total RNA (1–2 μg) was reverse transcribed to cDNA with a Reverse Trancription Kit (Applied Biosystems). Quantitative PCR was then performed in an AB7900-384 thermocycler (Applied Biosystem) using SYBR Green master mix (Applied Biosystems, Warrington, UK) as the reporter. Expression levels of target genes were normalized to the expression of housekeeping genes β-actin, GAPDH, β2-microglobulin and Yhwaz (tyrosine 3-monooxygenase/tryptophan 5-monooxygenase activation protein, ζ) for presentation of relative mRNA levels. Data were analysed with Biogazelle qBasePlus version 2.3 (Biogazelle) and graphs are represented as a normalized expression scaled to average of all samples. Gene-specific primers used are listed in S2 Table.

### Soluble embryonic alkaline phosphatase (SEAP)-NF-κB detection

50.000 transfected HEK-Blue hTLR2 cells with different HDAC6 constructions were place in bottom p96 well plates resuspended in HEK-Blue Detection medium (Invivogen) without stimulus (negative control) and with TLR-2 agonists (HKLM stimulus, MOI = 10). After 8–12 h of incubation, SEAP activity was measured by optical density at 620 nm with a microplate reader. To calculate the NF-κB induction, the signal obtained from each mutant condition without stimuli (background) was depleted of the signal of each condition of activation with Pam2GSK4, Pam3GSK4 or HKLM.

### ELISAs and nitrite measurement

Cytokine and NO production was analysed in the supernatants of BMDC cultures at 6, 12 and 24 h after stimulation with *Lm*, heat-killed *Listeria monocytogenes* (HKLM), heat-killed *Salmonella* Typhimurium (HKST), Pam3CSK4, Flagellin, Imiquimod, polyinosinic-polycytidylic acid (Poly(I:C)) (InvivoGen, San Diego, CA), or LPS from *Escherichia coli* (Sigma-Aldrich). TNF-α and IL12p70 were analysed with OptEIA ELISA kits (BD Biosciences, San Diego, CA), IL-1β and IL-6 with the mouse ELISA Ready-SET-Go! kit from eBioscience (Affymetrix, San Diego, CA) and Interferon-β was measured with Legend max mouse IFN-β ELISA kit (Biolegend). The detection was based on colorimetric quantification of absorbance at 450 nm, corrected with subtraction at 570 nm measured in a microplate reader (Bio-Rad Model 550). NO was estimated from the nitrite concentration measured with a Griess reagent kit at 548 nm (Molecular Probes/Life Technologies, Thermo Fisher Scientific). Results were expressed as the means of duplicate wells.

### Immunoblotting

Total cell extracts from BMDCs stimulated with *Lm*, HKLM or the indicated TLR ligands for the indicated times were prepared in lysis buffer (0.5% Triton X100, 25 mM Tris-HCl pH 7.5, 0.5 mM EGTA, 0.5 mM EDTA, 25 mM NaF, 0.5 sodium glycerol-phosphate, 2.5 mM pyrophosphate, 0.135 M sacarose) with a cocktail of protease and phosphatase inhibitors (Roche). Cell lysates were cleared of nuclei by centrifugation at 15,000 g for 15 min. Protein extracts were separated by 8–15% SDS-PAGE and transferred to a PVDF membrane (Biorad). Proteins were visualized with LAS-3000 after membrane incubation with specific antibodies (see S1 Table) and peroxidase-conjugated secondary antibodies (5 μg ml^−1^). Band intensities were quantified using Image Gauge software (Fuji Photo Film, Co., Ltd) and results are expressed relative to loading controls. For quantification of western-blots, phosphorylated/total ratios were divided by loading control signal. Non-infection (NI) time was considered as 100%, and following times were relativized to it.

### Immunoprecipitation of MyD88 and HDAC6 proteins

Human moDCs (1 × 10^7^ per condition) were lysed (10 mM Tris pH 7.4, 150 mM NaCl, 5% glycerol, 1mM EDTA, 1mM MgCl_2_, 1mM CaCl_2_, 1% CHAPS (Sigma) and protease and phosphatase inhibitors (Roche)) for 1 h at 4°C. Lysates were incubated for pre-clearing with pre-washed Protein G Dynabeads (Invitrogen; 50 μl per condition; 2 h, 4°C). Pre-cleared lysates were incubated with 6 μg rabbit anti-MyD88 antibody (Cell Signaling) or 6 μg rabbit anti-HDAC6 antibody (Assay bioTech) per condition O/N at 4°C. Similar μg of control isotype antibody for rabbit were used. Fifty microlitres of Dynabeads per condition were washed three times in wash buffer (10 mM Tris pH 7.4, 150 mM NaCl, 5% glycerol, 1mM EDTA, 1mM MgCl_2_, 1mM CaCl_2_, 0.1% CHAPS) and added to antibody-conjugated lysates for 2 h 4C. Antibody-conjugated Dynabeads were washed six times with wash buffer and transferred to clean tubes.

HEK293T cells or HEK-Blue hTLR2 (1 × 10^7^ per condition) were lysed (25 mM Tris pH 8, 150 mM NaCl, 0.5% NP-40 and protease and phosphatase inhibitors) and incubated for pre-clearing with pre-washed Protein G Dynabeads (Invitrogen; 50 μl per condition; 3 h, 4°C). Fifty microlitres of Dynabeads per condition were washed three times in wash buffer (25 mM Tris pH 8, 150 mM NaCl, 0.05% NP-40) and re-suspended in 600 μl of wash buffer containing 1–2 μg mouse anti-HA antibody (Roche) per condition and incubated 3 h at 4°C. Similar μg of control isotype antibody for mouse were used. Pre-cleared lysates were incubated with antibody-conjugated Dynabeads (O/N, 4°C). Antibody-conjugated Dynabeads were washed six times with lysis buffer and transferred to clean tubes. Then, were washed twice with wash buffer. Protein loading buffer was added, samples were boiled at 95°C for 5 min and processed for immunoblotting.

### Flow cytometry

Cells were stained in ice-cold PBS containing FBS (0.5%) and EDTA (5 mM) using appropriate antibody-fluorophore conjugates. Multiparameter analysis was performed on a FACSCANTO II flow cytometer (BD Biosciences) and analysed with FlowJo software (Tree Star). Prior to fixing, cells were resuspended in PBS/0.5% BSA/5 mM EDTA solution containing yellow fluorescent reactive dye to exclude dead cells (Life Technologies). For intracellular staining, cells were fixed and permeabilized using the CytoFix/Cytoperm kit (BD).

### Fluorescence confocal microscopy

For immunofluorescence assays, cells were plated onto slides coated with poly-L-lysine (50 μg ml^−1^) and incubated for 1 h at 37°C. Infection experiment were carried out at the indicated times. Cells were then fixed, blocked and stained with the indicated primary antibodies (5 μg ml^−1^) followed by alexa488- or Rhodamine Red X-labelled secondary antibodies (5 μg ml^−1^). Samples were examined under a Leica SP5 confocal microscope (Leica) fitted with a 63X objective. Images were acquired with sequential xyz acquisition mode scans with laser ranges of 418–473 nm for DAPI, 502–548 nm for Alexa-488, 584–644 nm for Rhodamine X and 737–779 nm for Alexa-647. Z-stacks of 2–5 μm were obtained using a maximum z-step size of 0.3 μm.

### Imaris quantification

Images were processed and assembled using Image J 1.51p (Fiji). Confocal 3D images assembled with Imaris 7.7.2 (Bitplane) using the ImarisCell module. Every cell and its corresponding intracellular bacteria were calculated in each image. Surfaces corresponding to bacteria were used to calculate the maximal fluorescence intensity of the channels to co-localize with bacteria. Two-channel co-localization was quantified in at least 10 images per genotype, corresponding to 10 biological samples.

### In-gel protein digestion

Proteins were in-gel digested using a previously described protocol [69]. Briefly, the coimmunoprecipitate was heated at 95°C for 5 min, after which the magnetic beads were removed using a magnet. The resulting solution was added sample buffer and loaded in 0.5-cm-wide wells of an SDS-PAGE gel. The run was stopped as soon as the front entered into the resolving gel. The protein band was visualized by Coomassie Blue staining, excised, and digested overnight at 37°C with 60 ng/μl sequencing-grade modified trypsin (Promega) at 10:1 protein:enzyme (w/w) ratio in 50 mM ammonium bicarbonate, pH 8.8, containing 10% acetonitrile. The resulting tryptic peptides were desalted onto C18 OMIX tips (Agilent), dried down and kept at -80°C until further use.

### Mass spectrometry

The resulting peptides were analyzed by liquid chromatography coupled to tandem mass spectrometry (LC-MS/MS) on an Easy nLC-1000 nano-HPLC apparatus (Thermo Scientific, San Jose, CA, USA) coupled to a hybrid quadrupole-orbitrap mass spectrometer (Q Exactive HF, Thermo Scientific). The dried peptides were taken up in 0.1% (v/v) formic acid and then loaded onto a PepMap100 C18 LC pre-column (75 μm I.D., 2 cm, Thermo Scientific) and eluted on line onto an analytical NanoViper PepMap 100 C18 LC column (75 μm I.D., 50 cm, Thermo Scientific) with a continuous gradient consisting of 8–31% B in 240 min (B = 80% ACN, 0.1% formic acid) at 200 nL/min. Peptides were ionized using a Picotip emitter nanospray needle (New Objective). Each MS run consisted of enhanced FT-resolution spectra (120,000 resolution) in the 400–1,200 m/z range followed by data-dependent MS/MS spectra of the 20 most intense parent ions acquired along the chromatographic run. The AGC target value in the Orbitrap for the survey scan was set to 1,000,000. Fragmentation in the linear ion trap was performed at 30% normalized collision energy with a target value of 10,000 ions. The full target was set to 30,000, with 1 microscan and 50 ms injection time, and the dynamic exclusion was set to 0.5 min.

### Peptide identification

For peptide identification the MS/MS spectra were searched with the Sequest algorithm implemented in Proteome Discoverer 1.4 (Thermo Scientific). Database searching against human protein sequences from the UniProt database (March 2017, 158,382 entries) was performed with the following parameters: trypsin digestion with 4 maximum missed cleavage sites; precursor and fragment mass tolerances of 800 ppm and 0.02 Da, respectively; Cys carbamidomethylation as static modifications; and Met oxidation and Lys acetylation as dynamic modifications. The results were analyzed using the probability ratio method [70] and a false discovery rate (FDR) for peptide identification was calculated based on the search results against a decoy database using the refined method [71].

### Statistical analysis

Data were analysed with GraphPad prism software (La Jolla, CA) for normality (Kolmogorov-Smirnov test for small samples). Normal data were analysed by Student t-test, non-normal data by Mann-Whitney test, and grouped data by 2-tailed One-way ANOVA with a Bonferroni post-test. For western blot quantification, the sample with the maximum signal was assigned a value of 100%, and signals in other samples were expressed as a percentage of this; significance was determined by a one-sample test. Long-rank (Mantel-Cox) test and Cehan-Breslow-Wilcoxon test were used for the analysis of the Kaplan-Meier curve (survival curve).

## Supporting information

S1 Fig Differentiation of GM-CSF-derived DCs, their viability at 6 hpi, comparison of CFUs of GM-CSF- and M-CSF-derived cells and fluorescent confocal microscopy of *Lm*. A) Left: Dot-plots showing CD11c and MHC-II markers, with gating for CD11c^+^MHC-II^+^ and CD11c^+^MHC-II^-^ populations (percentages indicated). Right: Dot-plots on differentiation day 11 showing FSC-H versus Gr-1, gating the Gr-1^+^ population corresponding to neutrophil contamination in GM-CSF-derived DC cultures. Charts show the percentages of CD11c^+^MHC-II^+^, CD11c^+^MHC-II^-^ and Gr-1^+^ populations. ns>0.05 non-significant; n = 6. B) Percentage viability of BMDCs before infections and at 6 hpi with *Lm*, ns>0.05 non-significant; n = 6. C) Comparison of CFUs in GM-CSF-derived DCs and M-CSF-derived macrophages over the time-course of *Lm* infection. ***p≤0.001, ** p≤0.01, ns>0.05 non-significant; n = 6. D) ImarisCell Module analysis of the number of cells and the number of bacteria per cell in all pictures (10 pictures per genotype). The graph shows the distribution of cells with a specific number of bacteria per cell. The number of cells with 6 and 7 bacteria differed significantly between the *Hdac6*^*+/+*^ and *Hdac6*^*-/-*^ genotypes. * p≤0.05, n = 10. E) Confocal microscopy determination of bacterial load of the Fig 1F. Maximum intensity z-projections of confocal microscopy images of *Lm*-infected *Hdac6*^*+/+*^ and *Hdac6*^*-/-*^ BMDCs at 6 hpi. ImarisCell Module view of the number of nucleus and bacteria per cell. Actin transparency is used to visualize bacteria (number indicated on the right). Images show DAPI (blue), *Lm* (red), β-actin (green). Scale bars 20 μm.
(TIF)

S2 Fig Gating strategy of myeloid populations of the spleen. A) Dot-plots showing the gating of myeloid populations of spleen. Dot-plots showing SSC-A versus FSC-A indicates p1, FSC-H versus FSC-W and SSC-H versus SSC-W were used to avoid doublets and FSC-H versus viability shows live and dead cells. Singlets and live cells were used to choose CD3^-^CD19^-^DX5^-^Ly6G^+^ cell population. From this population, neutrophils were gated as Ly6G+Ly6C+ cells, monocytes as CD11b^+^CD11c^lo^, Tips DCs as intermedium levels of CD11b and CD11c, conventional dendritic cells (cDCs) as CD11c^hi^; inside this population cDCs CD8^-^ were distinguish as CD11c^hi^CD11b^+^ and cDCs CD8^-^ as CD11c^hi^CD11b^lo^. B) Representative histograms of different splenic populations (monocytes, neutrophils, Tips DCs, total cDCs, cDCs CD8^-^ and cDCs CD8^+^) show *Lm* signal of *Hdac6*^*+/+*^ and *Hdac6*^*-/-*^ mice injected with a lethal dose of *Lm* at 6 hpi. A pool of *Hdac6*^*+/+*^ and *Hdac6*^*-/-*^ spleens non-infected was used as a control sample without infection (NI). **p≤0.01, * p≤0.05; n = 6.
(TIF)

S3 Fig Control vehicles and autophagy markers. A) Total CFUs at 0 and 6 hpi in *Lm*-infected BMDCs (MOI of 10) treated with different control vehicles (H_2_O, DMSO and ethanol). H_2_O were the control vehicle used for NH_4_Cl and cloroquine, DMSO for 3-MA, bafilomycin A1, DPI and 1400W and ethanol for rapamycin. Time 0 is included as a bacterial entry control. ***p≤0.001, ns>0.05 non-significant; n = 6. B) PCR analysis of autophagy markers (ATG-2, 5, 7 and 12, LC3A and B, p62 and Beclin-1) and lysosome markers (LAMP-1 and 2) (arbitrary units) after 6 hpi with *Lm*, ns>0.05 non-significant; n = 5.
(TIF)

S4 Fig Pro-inflammatory cytokine secretion. ELISA detection of the pro-inflammatory cytokines IL-1β and IL12p70 (pg/ml) in supernatants (S) and in supernatants plus the corresponding cell pellets (S+P) of *Lm*-infected *Hdac6*^*+/+*^ and *Hdac6*^*-/-*^ BMDCs at 6, 12 and 24 hpi. ***p≤0.001, ** p≤0.01, * p≤0.05, ns>0.05 non-significant; n = 5.
(TIF)

S5 Fig TLR expression and TLR-signalling pathway activation by LPS and HKLM. A) Western-blot analysis in *Hdac6*^*+/+*^ and *Hdac6*^*-/-*^ BMDCs over the time-course of LPS or HKLM treatment. Total and phosphorylated AKT were detected for both treatments. Accompanying charts on the right show quantification, indicating the percentage of phAKT/total AKT ratio. ** p≤0.01, * p≤0.05; n = 4. B) PCR analysis of TLR-1, 2 and 6 (arbitrary units) in *Hdac6*^*+/+*^ and *Hdac6*^*-/-*^ BMDCs non-infected (NI) and after *Lm*-infection at 6 hpi. ns>0.05 non-significant; n = 6.
(TIF)

S6 Fig Differentiation of FLT3-L DCs, their pro-inflammatory cytokine secretion at 6 hpi and association of HDAC6 with TLR-adaptor MyD88. A) Left: Dot-plots of FLT3-L DC cultures at day 11 of differentiation, showing gating for the CD11c+ population (percentages indicated). Centre: Dot-plots showing CD11b versus B220 to select two populations: CD11c^+^CD11b^+^B220^+^ (plasmacytoid DCs, pDCs) and CD11c^+^CD11b^+^B220^-^ (conventional DCs, cDCs) (percentages indicated). Right: Dot-plots showing CD11b versus CD24 to select the CD11b^+^CD24^+^ and CD11b^+^CD24^-^ populations (gated from cDCs) (percentages indicated). The charts on the right show the percentages of CD11c^+^, CD11c^+^CD11b^+^B220^-^CD24^-^ and CD11c^+^CD11b^+^B220^-^CD24^+^ populations, ns>0.05 non-significant; n = 6. B) ELISA detection of the pro-inflammatory cytokines IL-1β and IL-6 (pg/ml) in supernatants of *Hdac6*^*+/+*^ and *Hdac6*^*-/-*^ FLT3L-DCs activated with LPS, Imiquimod, Pam3GSK4, HKLM, HKST, *Lm*, Poly(I:C) or flagellin for 6 h. ***p≤0.001, ** p≤0.01, * p≤0.05; n = 6. C) MyD88 adaptor protein in *Hdac6*^*+/+*^ and *Hdac6*^*-/-*^ BMDCs. Western-blot analysis of MyD88 over the time-course of *Lm* infection in *Hdac6*^*+/+*^ and *Hdac6*^*-/-*^ BMDCs (left). Accompanying charts on the right show quantification of the percentage of MyD88; ns non-significant; n = 5. D) Immunoprecipitation of HA (MyD88) followed by western-blot for HDAC6 and MyD88. Immunoprecipitations were carried out using different HDAC6-eGFP plasmids co-transfected with MyD88-HA in HEK cell line. Over-expressed (HDAC6-eGFP, 160 kDa) is indicated at right of western-blot. E) Immunoprecipitation of HA (MyD88) followed by mass spectrometry analysis. Immunoprecipitations were carried out using different HDAC6-eGFP plasmids co-transfected with MyD88-HA in HEK cell line. The number of unique MyD88 and HDAC6 peptides identified is indicated. (*) indicates the presence of acetylated MyD88 peptides. Similar results were obtained in three independent experiments. F) MS^2^ fragmentation spectra from the peptides showing at 1217.0699 (Top), and 599.3803 (Bottom). Ion adscription to carboxy- (*y* ions, blue) and amino-terminal (*b* ions, red) fragmentation series is indicated. *K*_*ac*_ denotes acetylated lysine and *C*_*cm*_ indicates carbamidomethylated cysteine. Fragment ion sequence coverage is schematically indicated. Similar results were obtained in three independent experiments.
(TIF)

S1 Table Antibody table. Table of antibodies used in experimental procedures disclosed by reference, brand, host, application and dilution.
(PDF)

S2 Table qPCR primers. Table of qPCR primers used in experimental procedures disclosed by gene name and sequence 5´-3´.
(PDF)
