# Supplementary figures and images for: HDAC6 controls innate immune and autophagy responses to TLR-mediated signalling by the intracellular bacteria Listeria monocytogenes
Source: PLoS Pathog. 2017 Dec 27;13(12):e1006799. doi: 10.1371/journal.ppat.1006799 (PMC5760107; doi:10.1371/journal.ppat.1006799)

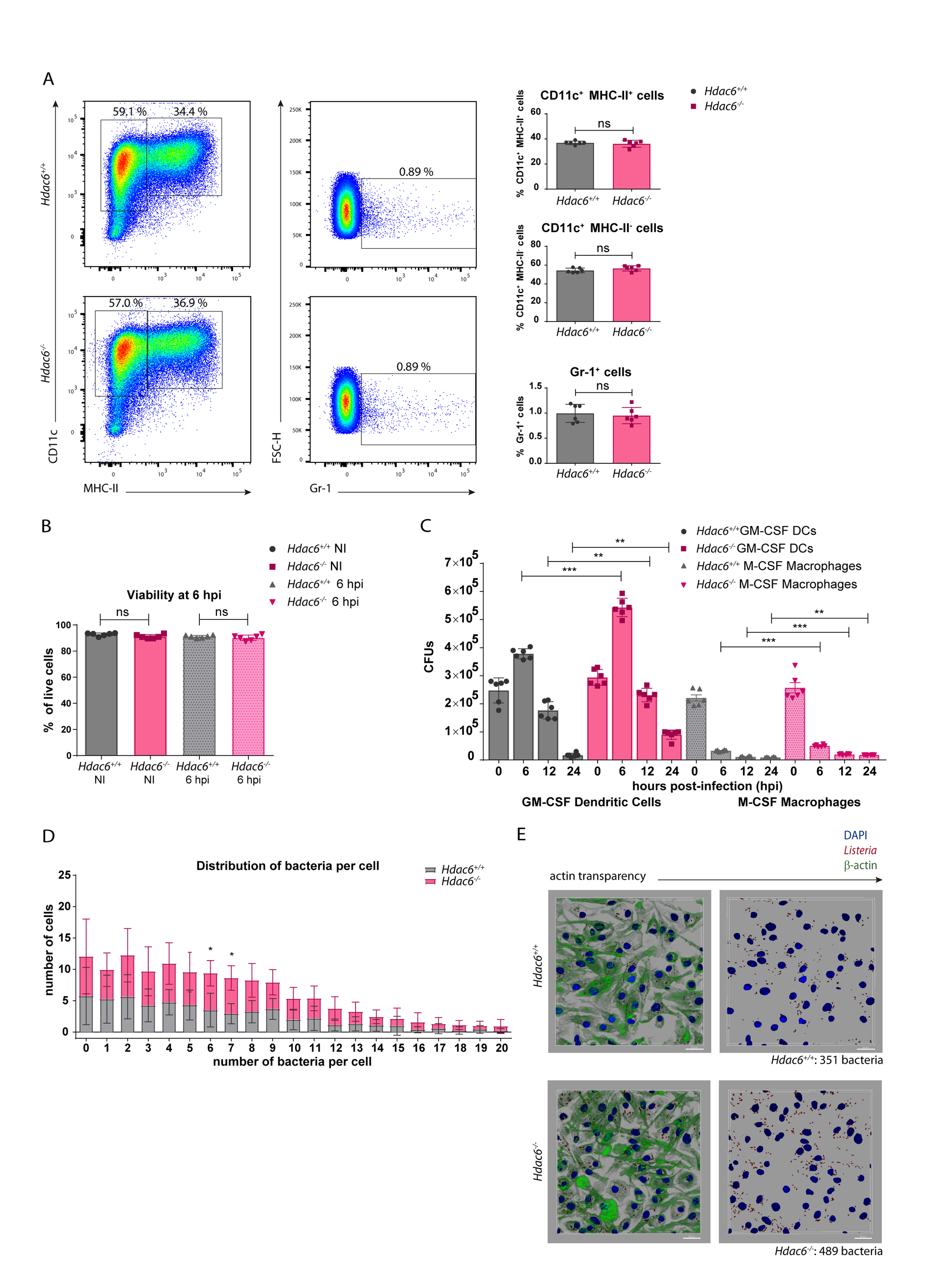

Supplement: S1 Fig — A) Left: Dot-plots showing CD11c and MHC-II markers, with gating for CD11c+MHC-II+ and CD11c+MHC-II- populations (percentages indicated). Right: Dot-plots on differentiation day 11 showing FSC-H versus Gr-1, gating the Gr-1+ population corresponding to neutrophil contamination in GM-CSF-derived DC cultures. Charts show the percentages of CD11c+MHC-II+, CD11c+MHC-II- and Gr-1+ populations. ns>0.05 non-significant; n = 6. B) Percentage viability of BMDCs before infections and at 6 hpi with Lm, ns>0.05 non-significant; n = 6. C) Comparison of CFUs in GM-CSF-derived DCs and M-CSF-derived macrophages over the time-course of Lm infection. ***p≤0.001, ** p≤0.01, ns>0.05 non-significant; n = 6. D) ImarisCell Module analysis of the number of cells and the number of bacteria per cell in all pictures (10 pictures per genotype). The graph shows the distribution of cells with a specific number of bacteria per cell. The number of cells with 6 and 7 bacteria differed significantly between the Hdac6+/+ and Hdac6-/- genotypes. * p≤0.05, n = 10. E) Confocal microscopy determination of bacterial load of the Fig 1F. Maximum intensity z-projections of confocal microscopy images of Lm-infected Hdac6+/+ and Hdac6-/- BMDCs at 6 hpi. ImarisCell Module view of the number of nucleus and bacteria per cell. Actin transparency is used to visualize bacteria (number indicated on the right). Images show DAPI (blue), Lm (red), β-actin (green). Scale bars 20 μm. (TIF) [file ppat.1006799.s001.tif]

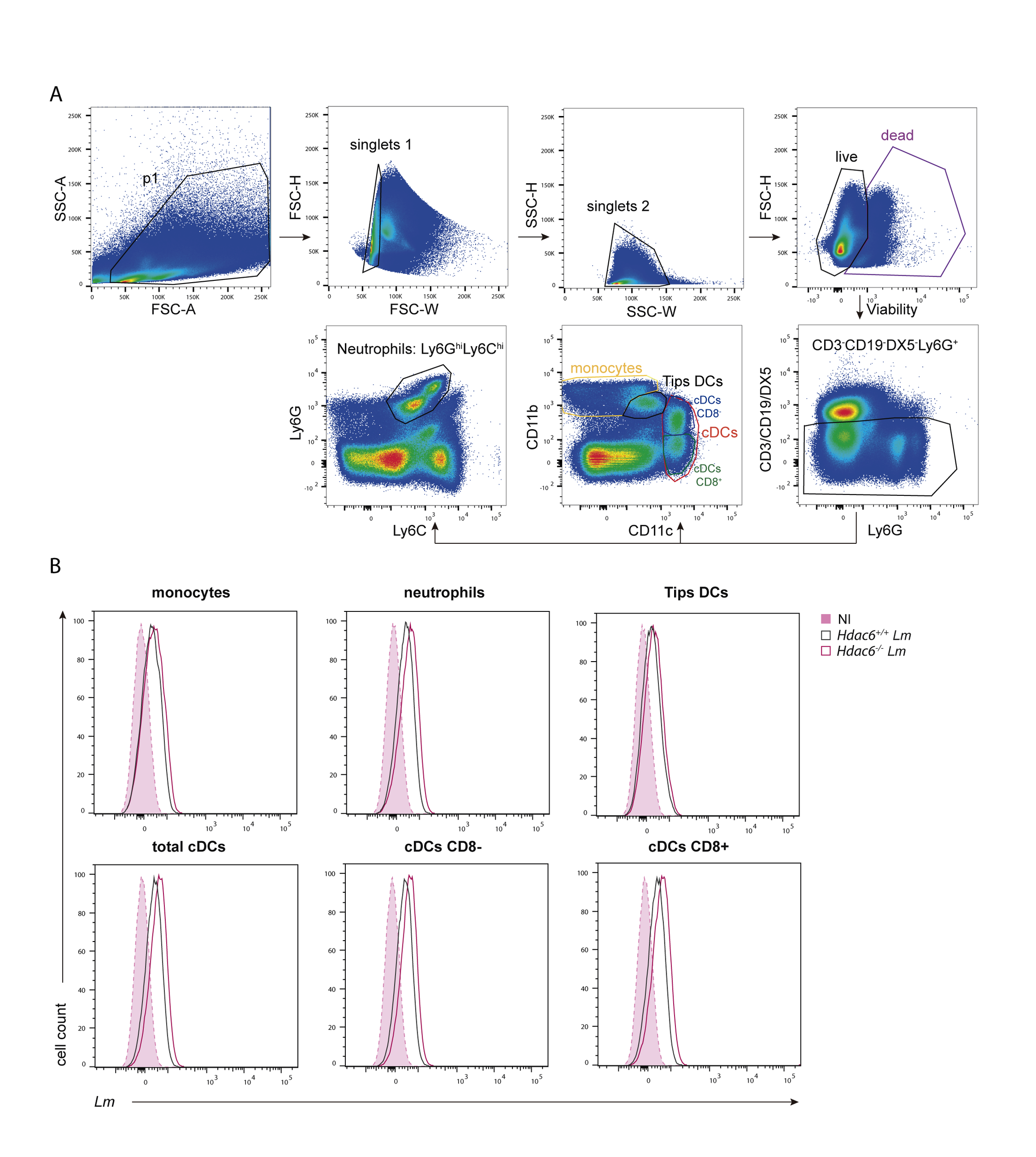

Supplement: S2 Fig — A) Dot-plots showing the gating of myeloid populations of spleen. Dot-plots showing SSC-A versus FSC-A indicates p1, FSC-H versus FSC-W and SSC-H versus SSC-W were used to avoid doublets and FSC-H versus viability shows live and dead cells. Singlets and live cells were used to choose CD3-CD19-DX5-Ly6G+ cell population. From this population, neutrophils were gated as Ly6G+Ly6C+ cells, monocytes as CD11b+CD11clo, Tips DCs as intermedium levels of CD11b and CD11c, conventional dendritic cells (cDCs) as CD11chi; inside this population cDCs CD8- were distinguish as CD11chiCD11b+ and cDCs CD8- as CD11chiCD11blo. B) Representative histograms of different splenic populations (monocytes, neutrophils, Tips DCs, total cDCs, cDCs CD8- and cDCs CD8+) show Lm signal of Hdac6+/+ and Hdac6-/- mice injected with a lethal dose of Lm at 6 hpi. A pool of Hdac6+/+ and Hdac6-/- spleens non-infected was used as a control sample without infection (NI). **p≤0.01, * p≤0.05; n = 6. (TIF) [file ppat.1006799.s002.tif]

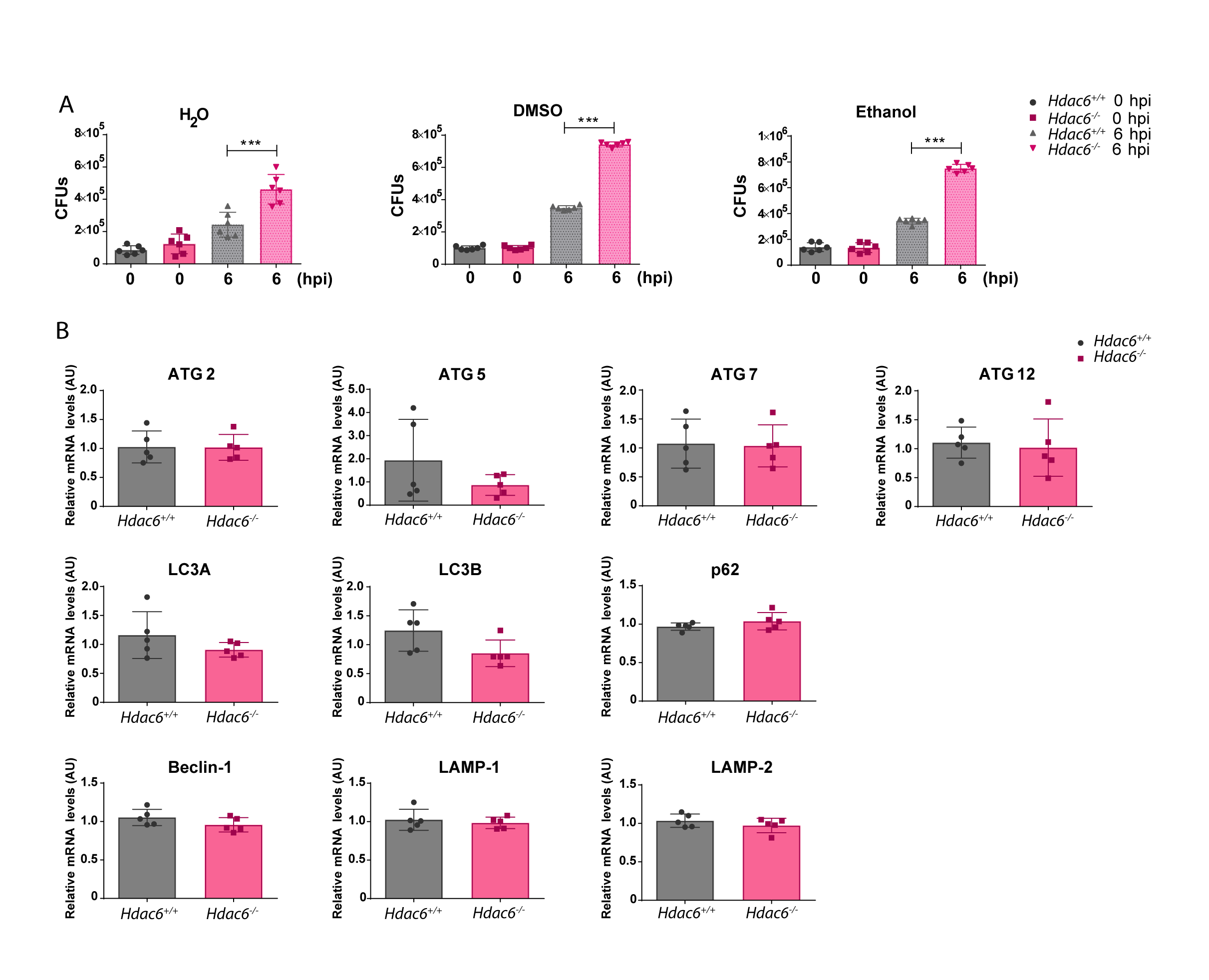

Supplement: S3 Fig — A) Total CFUs at 0 and 6 hpi in Lm-infected BMDCs (MOI of 10) treated with different control vehicles (H2O, DMSO and ethanol). H2O were the control vehicle used for NH4Cl and cloroquine, DMSO for 3-MA, bafilomycin A1, DPI and 1400W and ethanol for rapamycin. Time 0 is included as a bacterial entry control. ***p≤0.001, ns>0.05 non-significant; n = 6. B) PCR analysis of autophagy markers (ATG-2, 5, 7 and 12, LC3A and B, p62 and Beclin-1) and lysosome markers (LAMP-1 and 2) (arbitrary units) after 6 hpi with Lm, ns>0.05 non-significant; n = 5. (TIF) [file ppat.1006799.s003.tif]

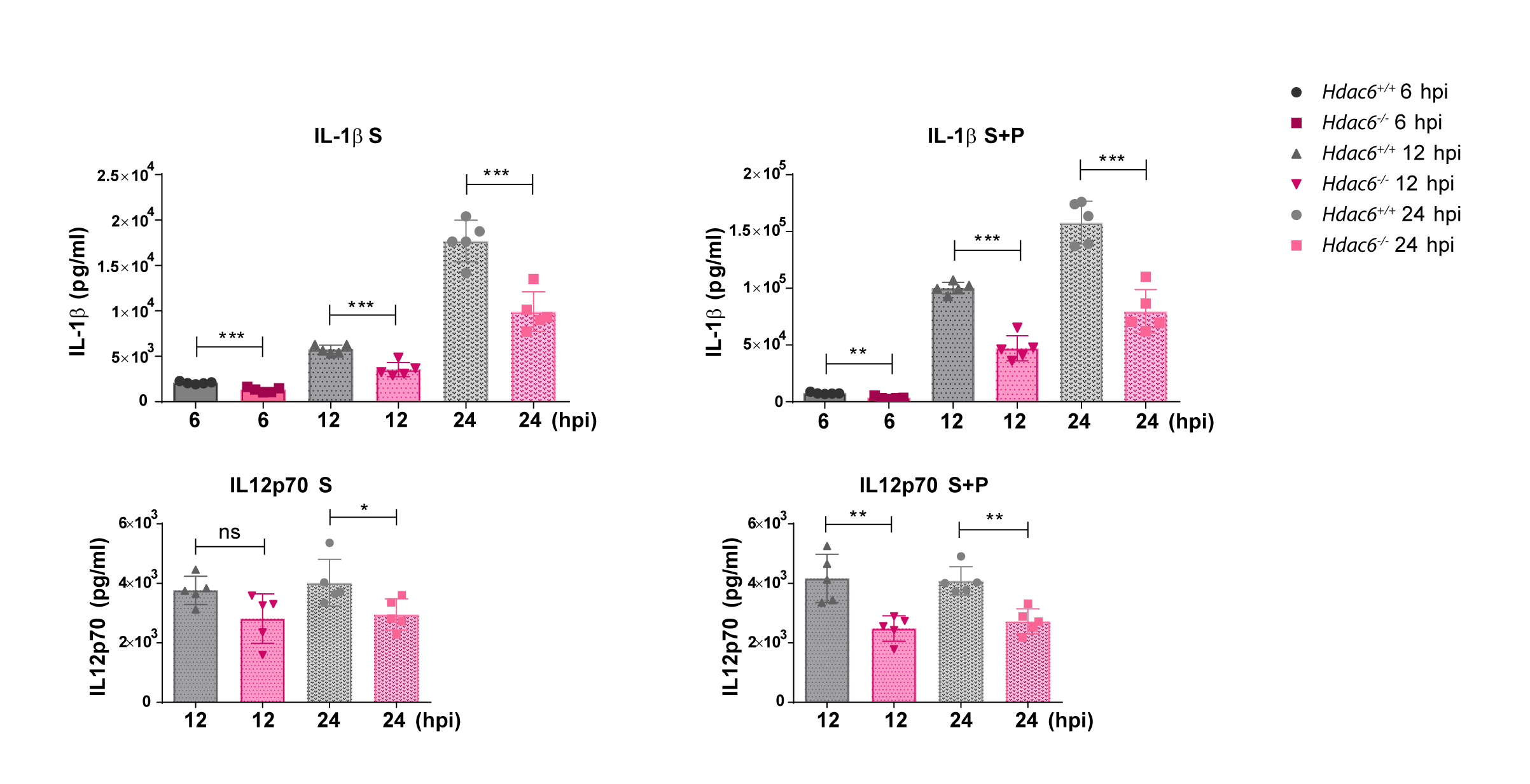

Supplement: S4 Fig — ELISA detection of the pro-inflammatory cytokines IL-1β and IL12p70 (pg/ml) in supernatants (S) and in supernatants plus the corresponding cell pellets (S+P) of Lm-infected Hdac6+/+ and Hdac6-/- BMDCs at 6, 12 and 24 hpi. ***p≤0.001, ** p≤0.01, * p≤0.05, ns>0.05 non-significant; n = 5. (TIF) [file ppat.1006799.s004.tif]

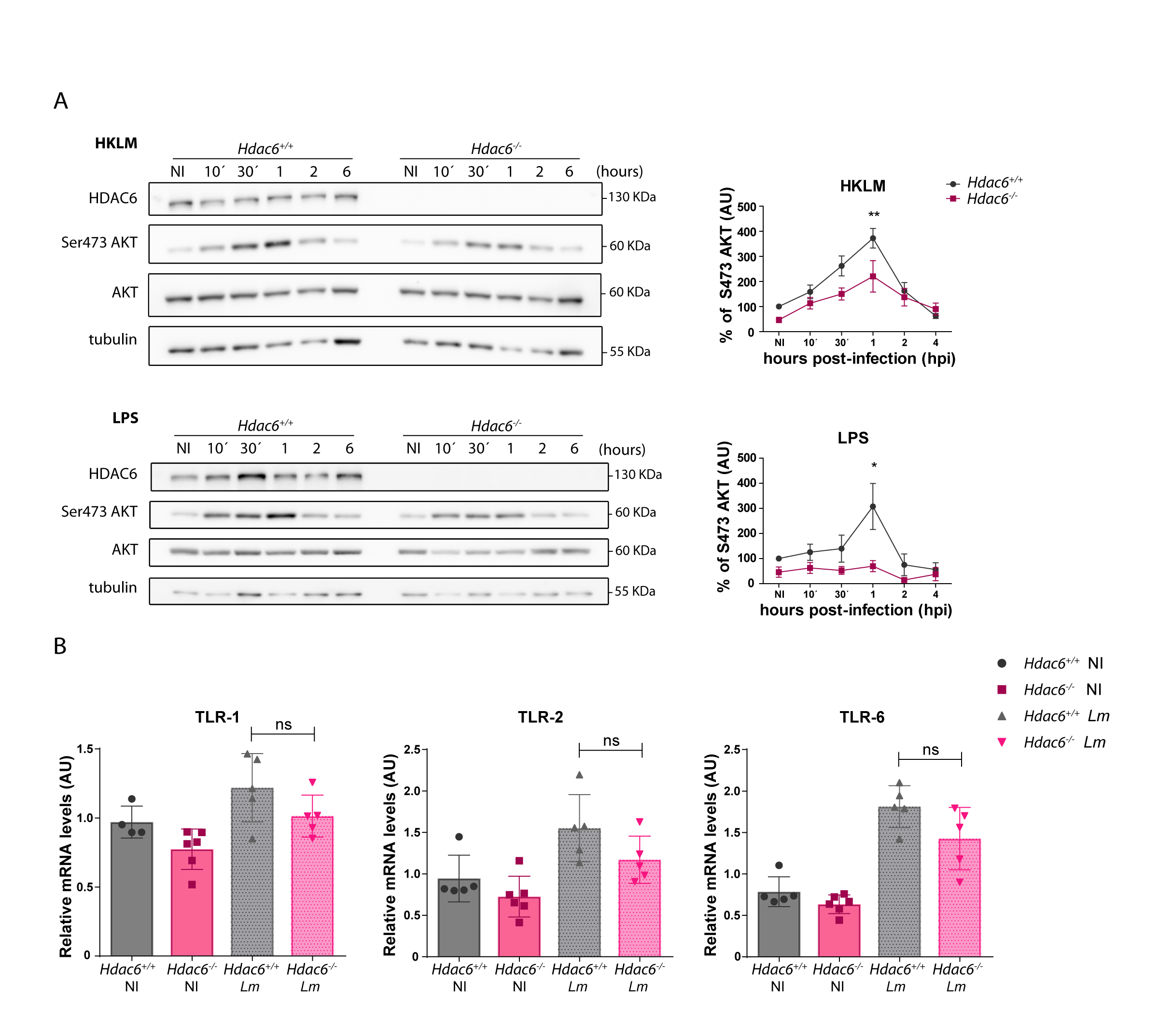

Supplement: S5 Fig — A) Western-blot analysis in Hdac6+/+ and Hdac6-/- BMDCs over the time-course of LPS or HKLM treatment. Total and phosphorylated AKT were detected for both treatments. Accompanying charts on the right show quantification, indicating the percentage of phAKT/total AKT ratio. ** p≤0.01, * p≤0.05; n = 4. B) PCR analysis of TLR-1, 2 and 6 (arbitrary units) in Hdac6+/+ and Hdac6-/- BMDCs non-infected (NI) and after Lm-infection at 6 hpi. ns>0.05 non-significant; n = 6. (TIF) [file ppat.1006799.s005.tif]

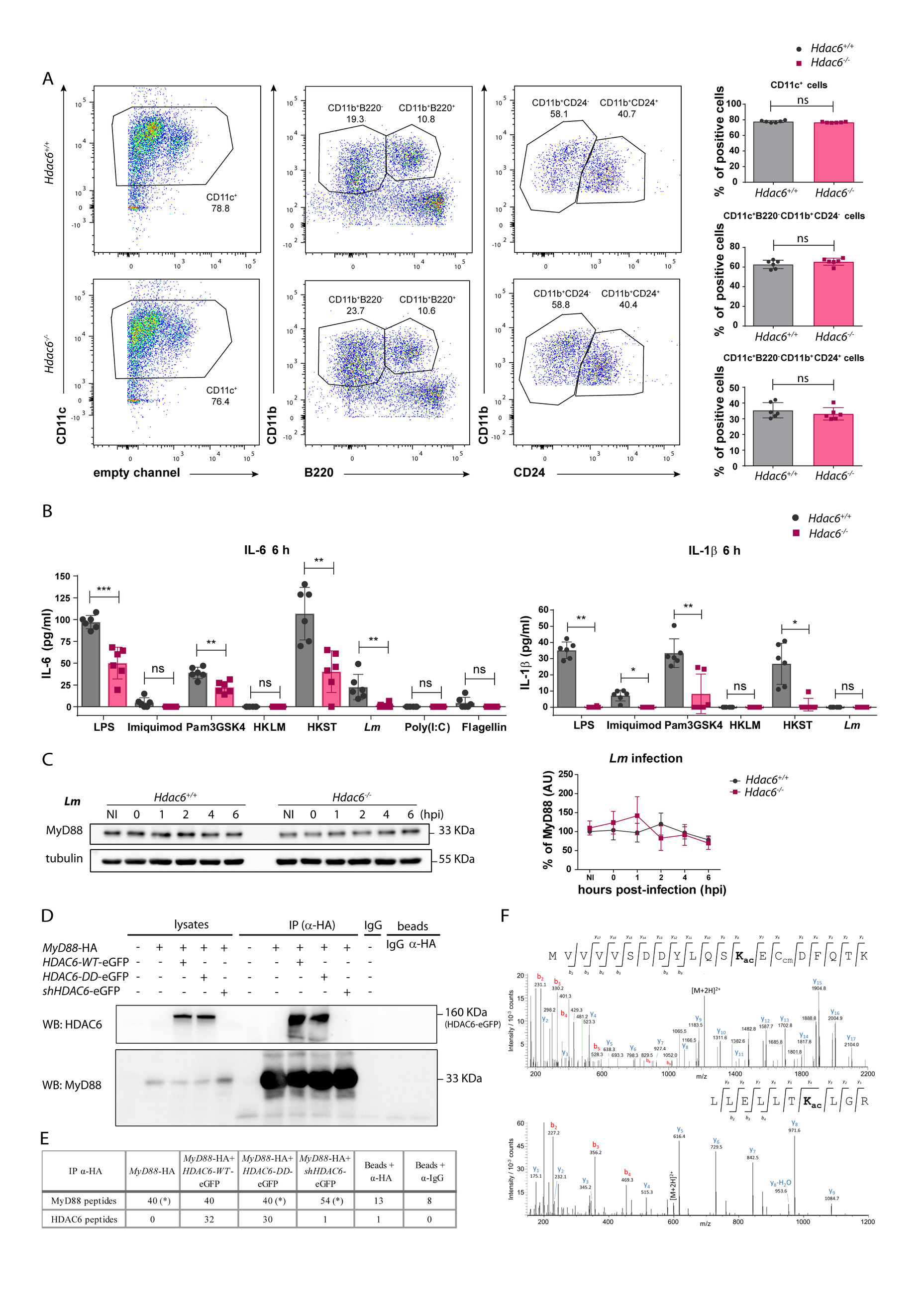

Supplement: S6 Fig — A) Left: Dot-plots of FLT3-L DC cultures at day 11 of differentiation, showing gating for the CD11c+ population (percentages indicated). Centre: Dot-plots showing CD11b versus B220 to select two populations: CD11c+CD11b+B220+ (plasmacytoid DCs, pDCs) and CD11c+CD11b+B220- (conventional DCs, cDCs) (percentages indicated). Right: Dot-plots showing CD11b versus CD24 to select the CD11b+CD24+ and CD11b+CD24- populations (gated from cDCs) (percentages indicated). The charts on the right show the percentages of CD11c+, CD11c+CD11b+B220-CD24- and CD11c+CD11b+B220-CD24+ populations, ns>0.05 non-significant; n = 6. B) ELISA detection of the pro-inflammatory cytokines IL-1β and IL-6 (pg/ml) in supernatants of Hdac6+/+ and Hdac6-/- FLT3L-DCs activated with LPS, Imiquimod, Pam3GSK4, HKLM, HKST, Lm, Poly(I:C) or flagellin for 6 h. ***p≤0.001, ** p≤0.01, * p≤0.05; n = 6. C) MyD88 adaptor protein in Hdac6+/+ and Hdac6-/- BMDCs. Western-blot analysis of MyD88 over the time-course of Lm infection in Hdac6+/+ and Hdac6-/- BMDCs (left). Accompanying charts on the right show quantification of the percentage of MyD88; ns non-significant; n = 5. D) Immunoprecipitation of HA (MyD88) followed by western-blot for HDAC6 and MyD88. Immunoprecipitations were carried out using different HDAC6-eGFP plasmids co-transfected with MyD88-HA in HEK cell line. Over-expressed (HDAC6-eGFP, 160 kDa) is indicated at right of western-blot. E) Immunoprecipitation of HA (MyD88) followed by mass spectrometry analysis. Immunoprecipitations were carried out using different HDAC6-eGFP plasmids co-transfected with MyD88-HA in HEK cell line. The number of unique MyD88 and HDAC6 peptides identified is indicated. (*) indicates the presence of acetylated MyD88 peptides. Similar results were obtained in three independent experiments. F) MS2 fragmentation spectra from the peptides showing at 1217.0699 (Top), and 599.3803 (Bottom). Ion adscription to carboxy- (y ions, blue) and amino-terminal (b ions, red) fragmentation [file ppat.1006799.s006.tif]
