# Supplementary material for: HDAC6 controls innate immune and autophagy responses to TLR-mediated signalling by the intracellular bacteria Listeria monocytogenes
Source: PLoS Pathog. 2017 Dec 27;13(12):e1006799. doi: 10.1371/journal.ppat.1006799 (PMC5760107; doi:10.1371/journal.ppat.1006799)
Supplement: S1 Table — Table of antibodies used in experimental procedures disclosed by reference, brand, host, application and dilution. (PDF) [file ppat.1006799.s007.pdf]

Supplemental Table 1. Antibody table.

Antibody	Reference	Brand	Host	Application	Dilution
Acetylated cortactin	09-881	Millipore	rabbit	IF	IF (1:100)
AKT	#9272	Cell Signaling	rabbit	WB	WB (1:1000)
Anti-HA	0000000115838 16001 (12CA5)	Roche (Sigma-Aldrich)	mouse	IP	IP (1-2 µg/point)
Anti-HA	A190-108A	Bethyl	rabbit	IP	IP (1-2 µg/point)
B220-PerCP-cyanine5.5	65-0452-U100 (RA 3-6B2)	Tombo biociences	rat	FC	FC (1:200)
CD11b-FITC	553310 (M1/70)	BD Pharmingen	rat	FC	FC (1:200)
CD11c-PE	557401 (HL3)	BD Pharmingen	hamster	FC	FC (1:200)
CD11c-PeCy7	558079 (HL3)	BD Pharmingen	hamster	FC	FC (1:200)
CD11c-PeCy7	55079	BD Pharmingen	hamster	FC	FC (1:200)
CD16/32 Fc block	(clone 2.4G)	BD Pharmingen	rat	FC	FC (1:200)
CD19- violetFluor450	75-0193-U100 (1D3)	Tombo biociences	rat	FC	FC (1:200)
CD24-PE	12-0241-82 (30-F1)	ebiocience	rat	FC	FC (1:200)
CD3-violetFluor450	75-0032-U100 (17A2)	Tombo biociences	rat	FC	FC (1:200)
CD8-APC	558079 (HL3)	BD Pharmingen	rat	FC	FC (1:200)
Chicken Anti-mouse 647	A-21463	Life technologies	chicken	FC	FC (1:500)
Chicken Anti-rabbit 647	A-21443	Life technologies	chicken	FC	FC (1:500)
Control isotype of mouse	sc-2025	Santa Cruz	rat	IP	IP (1-5 µg/point)
Control isotype of rabbit	sc-2027	Santa Cruz	rabbit	IP	IP (5 µg/point)
DX5 (CD49b)-V450	561638	BD biociences	rat	FC	FC (1:200)
ERK1/2	#9102	Cell Signaling	mouse	WB	WB (1:1000)
Goat anti rabbit 488 Hx	A-11008	Thermofisher Scientific	goat	IF	IF (1:500)
Goat anti rabbit Rodamin X HX	111-295-144	Jackson	goat	IF	IF (1:500)
Goat anti-mouse-HRP	#31430	Thermofisher Scientific	goat	WB	WB (1:5000)
Goat anti-rabbit-HRP	#31460	Thermofisher Scientific	goat	WB	WB (1:5000)
Gr-1-APC	553129 (RB6-8C5)	BD Pharmingen	rat	FC	FC (1:200)
HDAC6	C0226	Assay bioTech	rabbit	WB	WB (1:500)
iNOS	# 2982S	Cell Signaling	rabbit	WB	WB (1:500)
LC3b	# 2775S	Cell Signaling	rabbit	WB	WB (1:500)
Listeria	0400-0030	AbDSerotec	rabbit	FC	FC (1:200)
Ly6C-PerCP-Cy5.5	560525	Becton Dickinson	rat	FC	FC (1:200)
Ly6G-PE	551461 (1A8)	BD biociences	rat	FC	FC (1:200)
MHC-II I-A/I-E-APC	17-5321-81 (145/1/4.15.2)	ebiocience	rat	FC	FC (1:200)
MHC-II I-A/I-E-FITC	553623 (2G9)	BD Pharmingen	rat	FC	FC (1:500)
MyD88	D80F5 #4283	Cell Signaling	rabbit	WB	WB (1:500)
MyD88	sc-11356 (HFL-296)	Santa Cruz	rabbit	WB	WB(1:500)
MyD88	sc-136970 (B-1)	Santa Cruz	mouse	WB	WB(1:500)
MyD88	MA5-15762 (2E9C2)	TermoFisher Scientific	mouse	WB	WB (1:500)
p62	P0067	Sigma-Aldrich	rabbit	WB, FC	WB (1:1000); FC (1:200)

p70S6K	#2703	Cell Signaling	rabbit	WB	WB (1:1000)
phAKT (Ser473)	#9271	Cell Signaling	rabbit	WB	WB (1:1000)
phERK1/2 (Thr202/Thr204)	#9101	Cell Signaling	rabbit	WB	WB (1:1000)
php70S6K	#9205	Cell Signaling	rabbit	WB	WB (1:1000)
phS6 (Ser235/236)	#2211	Cell Signaling	rabbit	WB	WB (1:1000)
S6	#2717	Cell Signaling	rabbit	WB	WB (1:1000)
Streptavidin-APC	554067	BD Pharmingen	-	FC	FC (1:300)
tubulin	T9026 (DH1A)	Sigma-Aldrich	mouse	WB	WB (1:2000)
β-actin	A2228 (AC-74)	Sigma-Aldrich	mouse	WB	WB (1:2000)
HLA-DR-PerCP	560652 (G46-6)	BD biociences	mouse	FC	FC (1:200)
CD3e	T3b hybridoma	(68)	mouse	FC	FC (5 μg/ml)
DC-SIGN (CD209)	sc-59157 (MR-1)	Santa Cruz	mouse	FC	FC (1:200)
CD11c-PE	555392 (B-ly6)	BD biociences	mouse	FC	FC (1:200)
CD14-FITC	14F-100T (47-3D6)	Immunostep	mouse	FC	FC (1:200)
Streptavidin-BV421	563259	BD biociences	-	FC	FC (1:300)
