# Supplementary material for: HDAC6 controls innate immune and autophagy responses to TLR-mediated signalling by the intracellular bacteria Listeria monocytogenes
Source: PLoS Pathog. 2017 Dec 27;13(12):e1006799. doi: 10.1371/journal.ppat.1006799 (PMC5760107; doi:10.1371/journal.ppat.1006799)
Supplement: S2 Table — Table of qPCR primers used in experimental procedures disclosed by gene name and sequence 5´-3´. (PDF) [file ppat.1006799.s008.pdf]

Supplemental Table 2. qPCR primers.

Gene	Sequence 5´-3´
**Atg12 Fw**	AACAAAGAAATGGGCTGTGGAGCG
**Atg12 Rv**	TTCCGAGGCCACCAGTTTAAGGAA
**Atg2 Fw**	CCACCTCTGCAAATCGGCA
**Atg2 Rv**	CCAGTTGTCCTGATACCTCCA
**Atg5 Fw**	GACAAAGATGTGCTTCGAGATGTG
**Atg5 Rv**	GTAGCTCAGATGCTCGCTCAG
**Atg7 Fw**	ATGCCAGGACACCCTGTGAACTTC
**Atg7 Rv**	ACATCATTGCAGAAGTAGCAGCCA
**Beclin-1 Fw**	GGCCAATAAGATGGGTCTGA
**Beclin-1 Rv**	CACTGCCTCCAGTGTCTTCA
**CXCL10 Fw**	CCAAGTGCTGCCGTCATTTTC
**CXCL10 Rv**	TCCCTATGGCCCTCATTCTCA
**CXCL5 Fw**	TGCGTTGTGTTTGCTTAACCG
**CXCL5 Rv**	CTTCCACCGTAGGGCACTG
**CXCR1 Fw**	TCTGGACTAATCCTGAGGGTG
**CXCR1 Rv**	GCCTGTTGGTTATTGGAACTCTC
**IFIT3 Fw**	GTGGACTGAGATTTCTGAACTGC
**IFIT3 Rv**	CAGAGATTCCCGGTTGACCT
**IFN-β Fw**	TCAGAATGAGTGGTGGTTGC
**IFN-β Rv**	GACCTTTCAAATGCAGTAGATTCA
**IL12p40 Fw**	GGAAGCACGGCAGCAGAAT
**IL12p40 Rv**	AACTTGAGGGAGAAGTAGGAATGG
**IL-1β Fw**	GCAACTGTTCCTGAACTCAACT
**IL-1β Rv**	ATCTTTTGGGGTCCGTCAACT
**ISG15 Fw**	GGTGTCCGTGACTAACTCCAT
**ISG15 Rv**	TGGAAAGGGTAAGACCGTCCT
**LAMP-1 Fw**	AGCATACCGGTGTGTCAGTG
**LAMP-1 Rv**	GTTGGGGAAGGTCCATCCTG
**LAMP2a Fw**	TGGCTAATGGCTCAGCTTTC
**LAMP2a Rv**	ATGGGCACAAGGAAGTTGTC
**LC3A Fw**	TTGGTCAAGATCATCCGGC
**LC3A Rv**	GCTCACCATGCTGTGCTGG
**LC3B Fw**	CCCACCAAGATCCCAGTGAT
**LC3B Rv**	CCAGGAACTTGGTCTTGTCCA
**Mx1 Fw**	GACCATAGGGGTCTTGACCAA
**Mx1 Rv**	AGACTTGCTCTTTCTGAAAAGCC
**p62 Fw**	ATGTGGAACATGGAGGGAAGA
**p62 Rv**	GGAGTTCACCTGTAGATGGGT
**PanIFNα Fw**	CCTGAGARAGAAGAAACACAGCC
**PanIFNα Rv**	GGCTCTCCAGAYTTCTGCTCT
**TLR-1 Fw**	TGAGGGTCCTGATAATGTCCTAC
**TLR-1 Rv**	AGAGGTCCAAATGCTTGAGGC
**TLR-2 Fw**	GCAAACGCTGTTCTGCTCAG
**TLR-2 Rv**	AGGCGTCTCCCTCTATTGTATT
**TLR-6 Fw**	TGAGCCAAGACAGAAAACCCA
**TLR-6 Rv**	GGGACATGAGTAAGGTTCCTGTT
**TNF-α Fw**	CCCTTCCTCCGATGGCTAC
**TNF-α Rv**	CGCCTCCTTCTTGTTCTGG
